# Supplementary material for: Enhancing RNA editing efficiency and specificity with engineered ADAR2 guide RNAs
Source: Mol Ther Nucleic Acids. 2025 Jan 13;36(1):102447. doi: 10.1016/j.omtn.2025.102447 (PMC11834095; doi:10.1016/j.omtn.2025.102447)
Supplement: Document S1. Figures S1–S8 and Tables S1–S3 [file mmc1.pdf]

OMTN, Volume *36*

Supplemental information

Enhancing RNA editing efficiency and specificity with engineered ADAR2 guide RNAs

Xilei Ai, Sheng Ding, Shan Zhou, Feng Du, Shuai Liu, Xin Cui, Juan Dong, Xin Huang, and Zhuo Tang

Table S1: Guide sequence region of gRNAs.

Name	Sequence
1MS2-gRNA-dGFP	**cacgagggtgggcCagggcacg**ACATGAGGATCACCCATGT
1MS2-gRNA-dGFP (13nt blocker)	**GTGATCCTCATGTcacgagggtgggcCagggcacg**ACATGAGGATCACCCATGT
1MS2-gRNA-dGFP (15nt blocker)	**GGGTGATCCTCATGTcacgagggtgggcCagggcacg**ACATGAGGATCACCCATGT
1MS2-gRNA-dGFP (17nt blocker)	**ATGGGTGATCCTCATGTcacgagggtgggcCagggcacg**ACATGAGGATCACCCATGT
1MS2-gRNA-dGFP (19nt blocker)	**ACATGGGTGATCCTCATGTcacgagggtgggcCagggcacg**ACATGAGGATCACCCATGT
1MS2-gRNA-dGFP (21nt blocker)	**ACATGGGTGATCCTCATGTCGcacgagggtgggcCagggcacg**ACATGAGGATCACCCATGT
1MS2-gRNA-dGFP (23nt blocker)	**ACATGGGTGATCCTCATGTCGTGcacgagggtgggcCagggcacg**ACATGAGGATCACCCATGT
1MS2-gRNA-dGFP (25nt blocker)	**ACATGGGTGATCCTCATGTCGTGCCcacgagggtgggcCagggcacg**ACATGAGGATCACCCATGT
BSM-gRNA （15nt）-dGFP	**ACATGGGTGATCCTCATGTggtgggcCagggcac**ACATGAGGATCACCCATGT
BSM-gRNA （21nt）-dGFP	**ACATGGGTGATCCTCATGTgagggtgggcCagggcacggg**ACATGAGGATCACCCATGT
BSM-gRNA （25nt）-dGFP	**ACATGGGTGATCCTCATGTacgagggtgggcCagggcacgggca**ACATGAGGATCACCCATGT
BSM-gRNA （31nt）-dGFP	**ACATGGGTGATCCTCATGTgtcacgagggtgggcCagggcacgggcagct**ACATGAGGATCACCCATGT
1BoxB-gRNA-dGFP	**cacgagggtgggcCagggcacgGGCCCTGAAAAAGGGCC**
BSB-gRNA-dGFP (19nt blocker)	**ACATGGGTGATCCTCATGTcacgagggtgggcCagggcacgGGCCCTGAAAAAGGGCC**

1BoxB-gRNA- dGFP (2 mismatches)	**acgagggtaggcCagggtacgggcaGGCCCTGAAAAAGGGCC**
1BoxB-gRNA- dGFP (4 mismatches)	**acgagcgtaggcCagggtaccggcaGGCCCTGAAAAAGGGCC**
BSB-gRNA-dGFP (2 mismatches)	**ACATGGGTGATCCTCATGTacgagggtaggcCagggtacgggcaGGCCCTGAAAAAGGGCC**
BSB-gRNA-dGFP (4mismatches)	**ACATGGGTGATCCTCATGTacgagcgtaggcCagggtaccggcaGGCCCTGAAAAAGGGCC**
1BoxB-gRNA-ACTIN	**CctctcttgctcCgggcctcgtcgcGGCCCTGAAAAAGGGCC**
BSB-gRNA-ACTIN	**GGCCCTTTTTCAGGGCCGC**cctctcttgctcCgggcctcgtcgc**GGCCCTGAAAAAGGGCC**
1BoxB-gRNA-ACTIN (TCG)	**CcagttggtgacTatgccgtgctcgGGCCCTGAAAAAGGGCC**
BSB-gRNA-ACTIN (TCG)	**GGCCCTTTTTCAGGGCCCGccagttggtgacTatgccgtgctcgGGCCCTGAAAAAGGGCC**
1BoxB-gRNA-TYMS (TCG)	**TttcagtggctcTatgtgattcaggGGCCCTGAAAAAGGGCC**
BSB-gRNA-TYMS (TCG)	**GGCCCTTTTTCAGGGCCCCTttcagtggctcTatgtgattcaggGGCCCTGAAAAAGGGCC**

Table S2: Sequences of Constructs Used in this Study.

Name	Sequence
dGFP	ATGGTGAGCAAGGGCGAGGAGCTGTTCACCGGGGTGGTG<br>CCCATCCTGGTCGAGCTGGACGGCGACGTAAACGGCCAC<br>AAGTTCAGCGTGTCCGGCGAGGGCGAGGGCGATGCCACC<br>TACGGCAAGCTGACCCTGAAGTTCATCTGCACCACCGGC<br>AAGCTGCCCGTGCCC**TAG**CCCACCCTCGTGACCACCCTGA<br>CCTACGGCGTGCAGTGCTTCAGCCGCTACCCCGACCACAT<br>GAAGCAGCACGACTTCTTCAAGTCCGCCATGCCCGAAGG<br>CTACGTCCAGGAGCGCACCATCTTCTTCAAGGACGACGG<br>CAACTACAAGACCCGCGCCGAGGTGAAGTTCGAGGGCGA<br>CACCCTGGTGAACCGCATCGAGCTGAAGGGCATCGACTT<br>CAAGGAGGACGGCAACATCCTGGGGCACAAGCTGGAGTA<br>CAACTACAACAGCCACAACGTCTATATCATGGCCGACAA<br>GCAGAAGAACGGCATCAAGGTGAACTTCAAGATCCGCCA<br>CAACATCGAGGACGGCAGCGTGCAGCTCGCCGACCACTA<br>CCAGCAGAACACCCCCATCGGCGACGGCCCCGTGCTGCT

	GCCCGACAACCACTACCTGAGCACCCAGTCCGCCCTGAG<br>CAAAGACCCCAACGAGAAGCGCGATCACATGGTCCTGCT<br>GGAGTTCGTGACCGCCGCCGGGATCACTCTCGGCATGGA<br>CGAACTGTACAAGTAA
MCP-ADAR-NES	ATGGCTTCTAACTTTACGCAGTTTGTGCTCGTCGATAATG<br>GGGGAACAGGGGACGTGACAGTTGCCCCCTCAAACTTTG<br>CCAACGGCGTCGCTGAGTGGATTTCATCTAATTCCCGAAG<br>TCAGGCCTACAAGGTCACCTGCTCTGTTAGGCAAAGTTCA<br>GCCCAGAACCGCAAGTACACCATAAAGGTTGAAGTGCCC<br>AAGGTCGCGACCCAGACAGTCGGCGGAGTCGAACTGCCT<br>GTGGCTGCTTGGCGCTCATATCTCAATATGGAACTTACAA<br>TCCCGATCTTTGCAACTAATTCCGATTGTGAATTGATAGT<br>TAAGGCAATGCAGGGCTTGCTGAAGGATGGAAATCCGAT<br>CCCTTCAGCAATTGCCGCCAATAGCGGGATCTACGGATCC<br>GGAGGAGGTGGAAGCcagctgcatttaccgcaggttttagctgacgctgtctcac<br>gcctggtcctgggtaagtttggtgacctgaccgacaacttctcctcccctcacgctcgcagaaaa<br>gtgctggctggagtcgtcatgacaacaggcacagatgttaaagatgccaaggtgataagtgttt<br>ctacaggaacaaaatgtattaatggtgaatacatgagtgatcgtggccttgcattaaatgactgcc<br>atgcagaaataatatctcggagatccttgctcagatttctttatacacaacttgagctttacttaaata<br>acaaagatgatcaaaaaagatccatctttcagaaatcagagcgagggggggtttaggctgaagg<br>agaatgtccagtttcatctgtacatcagcacctctccctgtggagatgccagaatcttctcaccac<br>atgagccaatcctggaagaaccagcagatagacacccaaatcgtaaagcaagaggacagcta<br>cggaccaaaatagagtctggtCaggggacgattccagtgcgctccaatgcgagcatccaaac<br>gtgggacggggtgctgcaaggggagcggctgctcaccatgtcctgcagtgacaagattgcac<br>gctggaacgtggtgggcatccagggatcActgctcagcattttcgtggagcccatttacttctcg<br>agcatcatcctgggcagcctttaccacggggaccacctttccagggccatgtaccagcggatct<br>ccaacatagaggacctgccacctctctacaccctcaacaagcctttgctcagtggcatcagcaat<br>gcagaagcacggcagccagggaaggcccccaacttcagtgtcaactggacggtaggcgact<br>ccgctattgaggtcatcaacgccacgactgggaaggatgagctgggccgcgcgtcccgcctg<br>tgtaagcacgcgttgtactgtcgctggatgcgtgtgcacggcaaggttccctcccacttactacg<br>ctccaagattaccaagcccaacgtgtaccatgagtccaagctggcggcaaaggagtaccagg<br>ccgccaaggcgcgtctgttcacagccttcatcaaggcggggctgggggcctgggtggagaag<br>cccaccgagcaggaccagttctcactcacgGGGAGCGGATCCGGCTCTCT<br>GCCACCATTGGAAAGGCTTACGCTGTAA
4λN-ADAR-NES[1]	ATGGCGAACGCCAGAACCCGACGCAGAGAGAGGAGGGC<br>AGAGAAACAGGCCCAGTGGAAAGCCGCTAACGGAGGAG<br>GGGGATCCGGTGGAGGCGGCAGTGGAGGCGGGGGATCTA<br>ATGCGCGGACCCGCCGACGTGAACGGCGAGCAGAAAAAC<br>AGGCGCAATGGAAAGCAGCCAACGGCGGCGGTGGAAGT<br>GGAGGTGGTGGAAGCGGAGGCGGTGGTAGTAATGCCCGT<br>ACGCGCCGGCGCGAACGGCGAGCAGAAAAGCAGGCACA<br>GTGGAAGGCAGCTAATGGCGGAGGGGGAAGCGGGGGCG

	GTGGAAGTGGGGGTGGAGGCAGCAACGCACGAACACGA<br>CGACGTGAGCGTCGCGCTGAGAAACAAGCTCAATGGAAA<br>GCTGCAAACGGATCCGGAGGAGGTGGAAGCcagctgcatttaccg<br>caggttttagctgacgctgtctcacgcctggtcctgggtaagtttggtgacctgaccgacaacttc<br>tcctcccctcacgctcgcagaaaagtgctggctggagtcgtcatgacaacaggcacagatgtta<br>aagatgccaaggtgataagtgtttctacaggaacaaaatgtattaatggtgaatacatgagtgatc<br>gtggccttgcattaaatgactgccatgcagaaataatatctcggagatccttgctcagatttctttat<br>acacaacttgagctttacttaaataacaaagatgatcaaaaaagatccatctttcagaaatcagag<br>cgaggggggtttaggctgaaggagaatgtccagtttcatctgtacatcagcacctctccctgtgg<br>agatgccagaatcttctcaccacatgagccaatcctggaagaaccagcagatagacacccaaa<br>tcgtaaagcaagaggacagctacggaccaaaatagagtctggtCaggggacgattccagtgc<br>gctccaatgcgagcatccaaacgtgggacggggtgctgcaaggggagcggctgctcaccat<br>gtcctgcagtgacaagattgcacgctggaacgtggtgggcatccagggatcActgctcagcat<br>tttcgtggagcccatttacttctcgagcatcatcctgggcagcctttaccacggggaccacctttc<br>cagggccatgtaccagcggatctccaacatagaggacctgccacctctctacaccctcaacaa<br>gcctttgctcagtggcatcagcaatgcagaagcacggcagccagggaaggcccccaacttca<br>gtgtcaactggacggtaggcgactccgctattgaggtcatcaacgccacgactgggaaggatg<br>agctgggccgcgcgtcccgcctgtgtaagcacgcgttgtactgtcgctggatgcgtgtgcacg<br>gcaaggttccctcccacttactacgctccaagattaccaagcccaacgtgtaccatgagtccaa<br>gctggcggcaaaggagtaccaggccgccaaggcgcgtctgttcacagccttcatcaaggcgg<br>ggctgggggcctgggtggagaagcccaccgagcaggaccagttctcactcacgGGGAG<br>CGGATCCGGCTCTCTGCCACCATTGGAAAGGCTTACGCTG
4λN-ADAR$_{\text{RECUSE}}$-NES^{2} ADAR$_{\text{RESCUE}}$ contains mutations of ADAR2(E488Q/V351G/S486A/T375S/S370C/P462A/N597I/L332I/I398V/K350I/M383L/D619G/S582T/ V440I/ S495N/ K418E	ATGGCGAACGCCAGAACCCGACGCAGAGAGAGGAGGGC<br>AGAGAAACAGGCCCAGTGGAAAGCCGCTAACGGAGGAG<br>GGGGATCCGGTGGAGGCGGCAGTGGAGGCGGGGGATCTA<br>ATGCGCGGACCCGCCGACGTGAACGGCGAGCAGAAAAAC<br>AGGCGCAATGGAAAGCAGCCAACGGCGGCGGTGGAAGT<br>GGAGGTGGTGGAAGCGGAGGCGGTGGTAGTAATGCCCGT<br>ACGCGCCGGCGCGAACGGCGAGCAGAAAAGCAGGCACA<br>GTGGAAGGCAGCTAATGGCGGAGGGGGAAGCGGGGGCG<br>GTGGAAGTGGGGGTGGAGGCAGCAACGCACGAACACGA<br>CGACGTGAGCGTCGCGCTGAGAAACAAGCTCAATGGAAA<br>GCTGCAAACGGATCCGGAGGAGGTGGAAGCcagctgcatttaccg<br>caggttttagctgacgctgtctcacgcCTGgtcATAggtaagtttggtgacctgaccgaca<br>acttctcctcccctcacgctcgcagaaTaGGTctggctggagtcgtcatgacaacaggcaca<br>gatgttaaagatgccAAGgtgataTGTgtttctacaggaTCTaaatgtattaatggtgaat<br>acCTAagtgatcgtggccttgcattaaatgactgccatgcagaaataGtatctcggagatcctt<br>gctcagatttctttatacacaacttgagctttacttaaataacGAGgatgatcaaaaaagatccat<br>ctttcagaaatcagagcgaggggggtttaggctgaaggagaatATAcagtttcatctgtacat<br>cagcacctctccctgtggagatgccagaatcttctcaccacatgagGCAatcctggaagaac<br>cagcagatagacacccaaatcgtaaagcaagaggacagctacggaccaaaatagagGCTg<br>gtCaggggacgattccaGTGcgcAACaatgcgagcatccaaacgtgggacggggtgct<br>gcaaggggagcggCTGctcaccatgtcctgcagtgacaagattgcacgctggaacgtggtg

	ggcatccagggatcActgctcagcattttcgtggagcccatttacttctcgagcatcatcctggg<br>cagcctttaccacggggaccaccttTCCagggccatgtacCAGcggatctccaacataga<br>ggacctgccacctctctacaccctcaacaagcctttgctcACAggcatcagcaatgcagaag<br>cacggcagccagggaaggcccccATAttcagtgtcaactggacggtaggcgactccgcta<br>ttgaggtcatcaacgccacgactgggaagGGAGAGctgggccgcgcgtcccgcctgtgt<br>aagcacgcgttgtactgtcgctggatgcgtgtgcacggcaaggttccctcccacttaCTAcgc<br>tccaagattaccaagcccaacgtgtaccatgagACAaagctggcggcaaaggagtaccag<br>gccgccaaggcgcgtctgttcacagccttcatcaaggcggggctgggggcctgggtggagaa<br>gcccaccgagcaggaccagttctcactcacgGGGAGCGGATCCGGCTCTCT<br>GCCACCATTGGAAAGGCTTACGCTGTAA

Table S3: PCR, and Sequencing primers.

Name	Sequence
GFP-PCR-F	GGGCGAGGAGCTGTTCACC
GFP-PCR-R	GCTGCCGTCCTCGATGTTG
GAPDH-PCR-F	GAAATCCCATCACCATCTTCC
GAPDH-PCR-R	GGTCTCTCTCTTCCTCTTGT
Actin-PCR-F	ATGGATGATGATATCGCCGC
Actin-PCR-R	GAGGCGTACAGGGATAGCACA
TYMS-PCR-F	CAGGACAGGGAGTTGACCAA
TYMS-PCR-R	GTCTTTAGGGGTTGGGCTGG

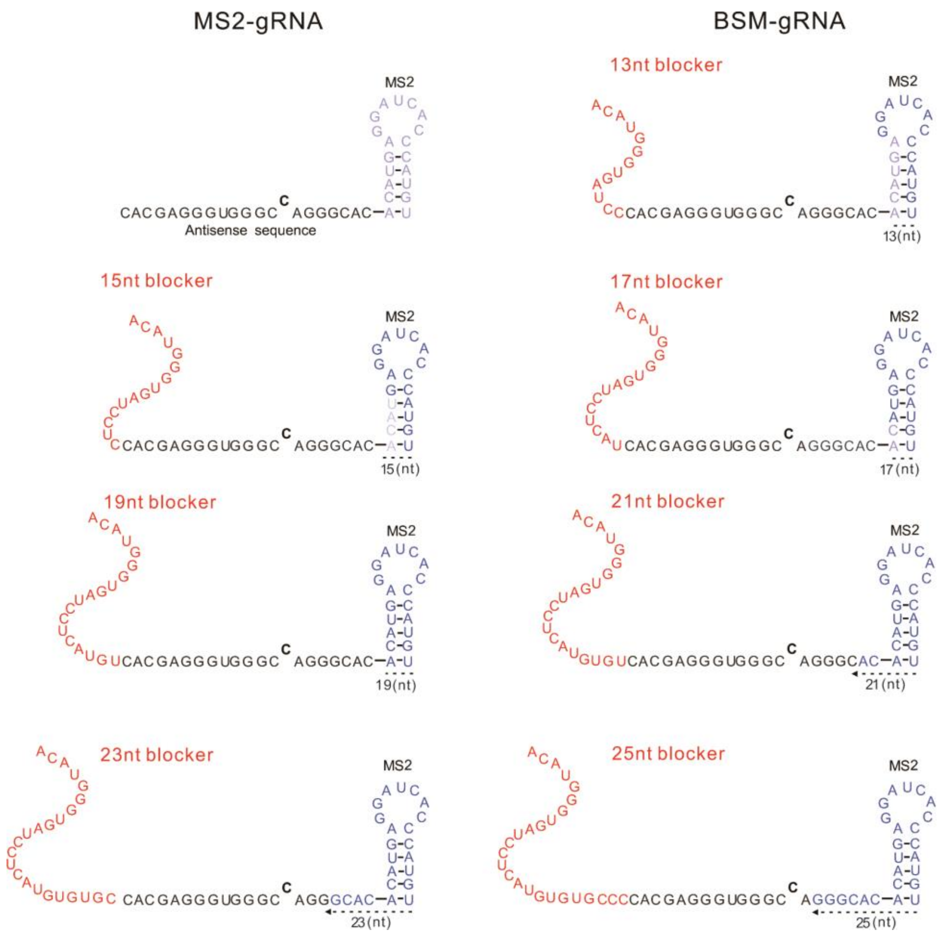

Figure S1. The schematic diagram of MS2-gRNA and BSM-gRNA with different blocking sequence lengths. As shown in the figure below, all blocking sequences (red) and their complementary sequences (blue) used in the BSM-gRNA design are displayed. When the blocking sequence exceeded the length of the MS2 hairpin (19 nt), extending the blocking sequence by targeting regions near the MS2 hairpin, gradually elongating it from the 3' to the 5' end. This design was also applied to the BoxB system.

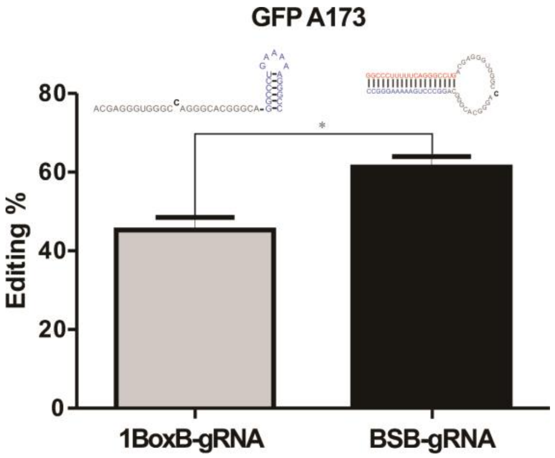

Figure S2. 1BoxB-gRNA and BSB-gRNA mediated editing of dGFP A173. ADAR-mediated editing efficiencies with the addition of plasmids expressing 1BoxB-gRNA, BSB-gRNA that target the same site (dead-GFP). HEK293 cells were cultured in 24-well plates. 500ng of pcDNA3.1-1BoxB-λN-ADAR (or pcDNA3.1-BSB-λN-ADAR) and 500 ng pcDNA3.1-dead GFP report plasmids were transfected when cells grow to approximately 70–80% confluence. All transfections were conducted using Hieff Tran^{TM} Liposomal Transfection Reagent (Yeasen) according to the manufacturer's recommendations. The transformed media was changed at 24 hours and the cells were incubated for additional 48 hours before analysis. Finally, the targeted RNA editing was confirmed by RT-PCR/Sanger sequencing. The blocking sequence containing BoxB-gRNA was paired with the target RNA of dead-GFP, and the results showed a 1.4-fold increase in BSB-gRNA over BoxB-gRNA to 61%. All values are mean ± SEM with n = 3 biological replicates. Student t-test: **P* <0.05, ***P* <0.01, ****P* <0.001.

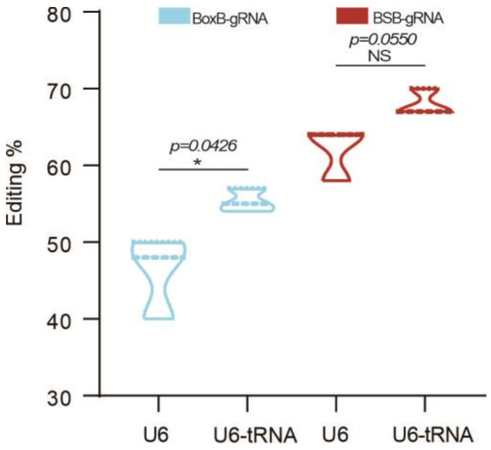

Figure S3. Effect of U6 and U6-tRNA promoters on RNA editing efficiency in the SPRING system using BoxB-gRNA and BSB-gRNA. The figure shows the RNA editing efficiencies of BoxB-gRNA and BSB-gRNA driven by U6 and U6-tRNA promoters in the SPRING system. The U6-tRNA promoter significantly improved editing efficiency for BoxB-gRNA, increasing from 62% (U6) to 68% (P = 0.0426, * indicates statistical significance). However, for BSB-gRNA, the increase was not statistically significant (P = 0.0550, NS indicates not significant).

Note: Solid lines represent the median, and dashed lines denote the first and third quartiles. Statistical analysis was performed using a two-tailed Student's t-test..

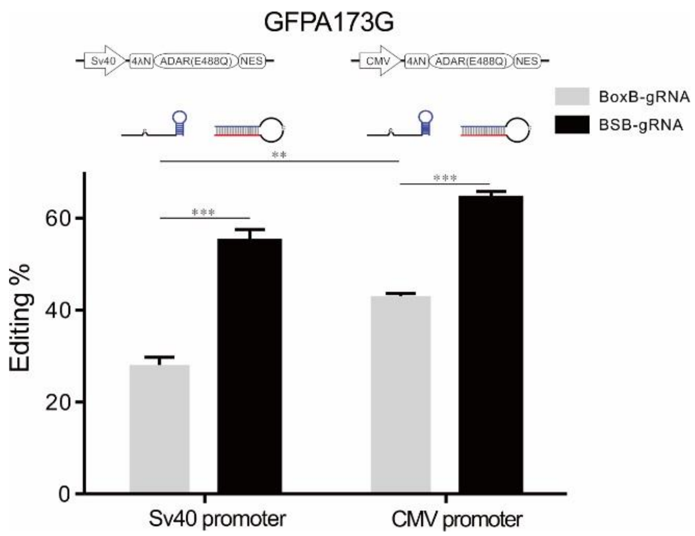

Figure S4. **The SV40 promoter and the CMV promoter respectively mediate RNA editing.** To further validate this mechanism, we transcribed the effector 4λN-ADAR(E488Q) using two different promoters (SV40 and CMV). ADAR-mediated editing efficiencies with the addition of plasmids expressing 1BoxB-gRNA, BSB-gRNA that target the same site (dead-GFP). HEK293 cells were cultured in 24-well plates. 500ng of pcDNA3.1-1BoxB-λN-ADAR (or pcDNA3.1-BSB-λN-ADAR) and 500 ng pcDNA3.1-dead GFP report plasmids were transfected when cells grow to approximately 70–80% confluence. All transfections were conducted using Hieff Tran^{TM} Liposomal Transfection Reagent (Yeasen) according to the manufacturer's recommendations. The transformed media was changed at 24 hours and the cells were incubated for additional 48 hours before analysis. Finally, the targeted RNA editing was confirmed by RT-PCR/Sanger sequencing. The editing efficiency of dGFP 173A site BoxB-gRNA transcribed by the SV40 promoter was 28%, compared to 55% for 1BoxB-gRNA, representing a 49% increase. For the CMV promoter-transcribed effector, BoxB-gRNA editing efficiency was 64%, compared to 43% for 1BoxB-gRNA, resulting in a 33% improvement. All values are mean ± SEM with n = 3 biological replicates. Student t-test: $*P < 0.05$, $**P < 0.01$, $***P < 0.001$.

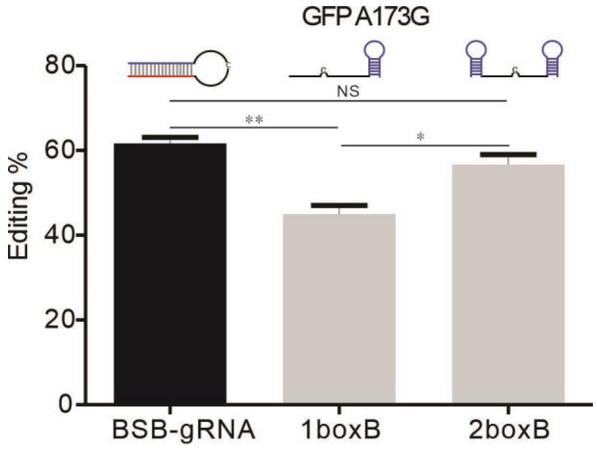

Figure S5. **Comparison of SPRING system with different numbers of BoxB guides.** Furthermore, we conducted a comparative analysis of RNA editing utilizing our system alongside the guide RNA containing two BoxB aptamers positioned towards both termini of the antisense part. HEK293 cells were cultured in 24-well plates. 500ng of pcDNA3.1-1BoxB-λN-ADAR (or pcDNA3.1-BSB-λN-ADAR) and 500 ng pcDNA3.1-dead GFP report plasmids were transfected when cells grow to approximately 70–80% confluence. All transfections were conducted using Hieff Tran^{TM} Liposomal Transfection Reagent (Yeasen) according to the manufacturer's recommendations. The transformed media was changed at 24 hours and the cells were incubated for additional 48 hours before analysis. Finally, the targeted RNA editing was confirmed by RT-PCR/Sanger sequencing.
At the dGFP 173A site, the editing efficiency of 2BoxB-gRNA was 57%, higher than the 45% efficiency of 1BoxB-gRNA, while the editing efficiency of BSB-gRNA at this site was 62%, surpassing the editing efficiencies of both at this location. All values are mean ± SEM with n = 3 biological replicates. Using one-way ANOVA revealed significant differences among the three groups (F=18.99,P=0.0025,R^2=0.8636). Tukey's post hoc test was performed to identify pairwise differences: BSB-gRNA *vs.* 1BoxB-gRNA (*P*=0.0023,＊＊), BSB-gRNA *vs.* 2BoxB-gRNA (*P*=0.2473,ns), 1BoxB-gRNA *vs.* 2BoxB-gRNA (*P*=0.0134,*). Significance thresholds: **P*<0.05,** *P*<0.01,****P*<0.001.

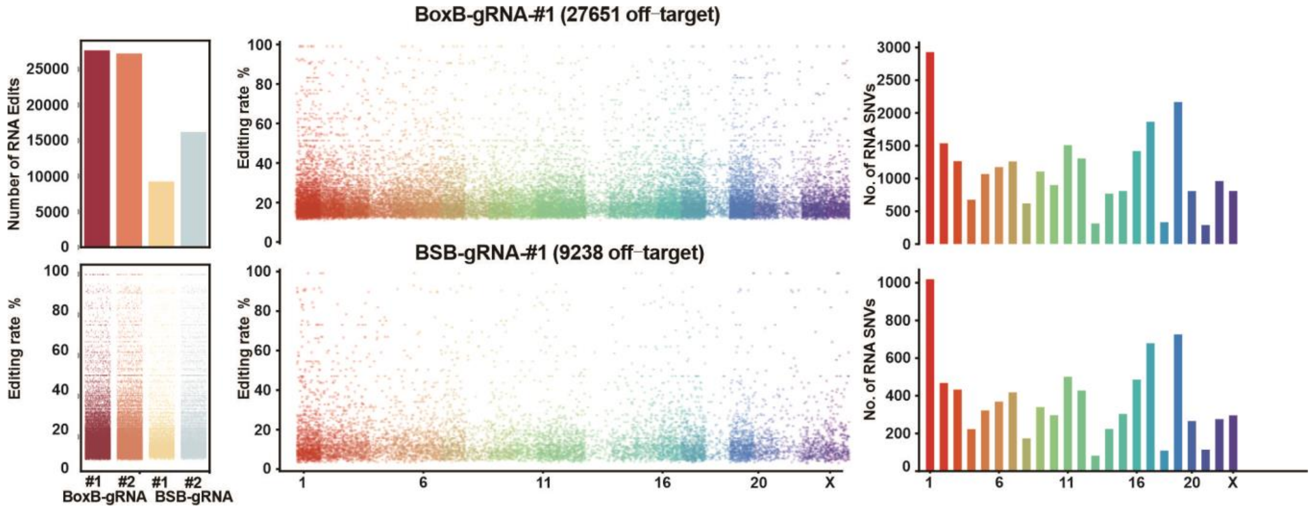

Figure S6. Analysis of transcriptome-wide off-target editing in the BoxB-gRNA group and BSB-gRNA group. Furthermore, we sought to evaluate the specificity of BSB-gRNAs both at the transcriptome-wide level and for target transcripts. HEK293T cells were cultured in 6-well plates, and 2500 ng of pcDNA3.1-4λN-DD-1BoxB-gRNA (or pcDNA3.1-4λN-DD-BSB-gRNA) plasmids were transfected when cells reached approximately 70–80% confluence. All transfections were performed using Hieff-Tran^{TM} Liposomal Transfection Reagent (Yeasen), following the manufacturer's instructions. The media were replaced after 24 hours, and the cells were incubated for an additional 48 hours prior to analysis. To assess transcriptome-wide specificity, deep RNA-seq analysis was conducted on samples transfected with BoxB-gRNA or BSB-gRNA, as well as an untransfected HEK293T control. The results revealed that compared to BoxB-gRNA, which exhibited nearly 30,000 off-target sites, BSB-gRNA demonstrated a substantial reduction, with approximately 12,000 off-target sites. These findings highlight the enhanced efficiency and specificity of the SPRING system, surpassing the performance of the original BoxB-λN-ADAR system (n=2; p=0.0259; unpaired t-test, one-tailed).

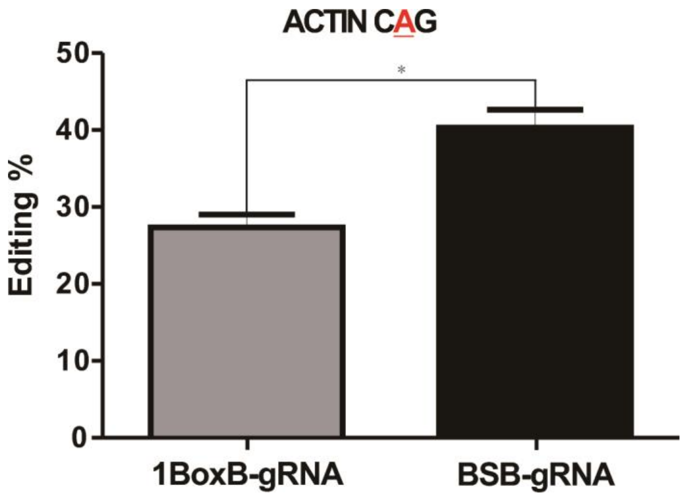

Figure S7. **1BoxB-gRNA and BSB-gRNA mediated editing of CAG site in the ORF of ACTIN.** We next sought to assess their editing efficiency in endogenous transcript editing. We selected a UAG editing site within the open reading frame (ORF) of ACTIN, which is usually the target of other RNA base editing systems. We introduced 4λNs-DD and BSB-gRNA into the cells and aligned the gRNAs along the endogenous transcripts. Analysing the editing of the endogenous RNAs by RT-PCR/Sanger sequencing, we found that the peak of editing appeared 48 hours after transfection. The level of A-I RNA editing increased from 27% to 40% at specific sites of the ACTIN gene. In conclusion, these results confirm that BSB-gRNA can effectively improve the efficiency of ADAR-mediated RNA editing. All values are mean ± SEM with n = 3 biological replicates. Student t-test: **P* <0.05, ***P* <0.01, ****P* <0.001.

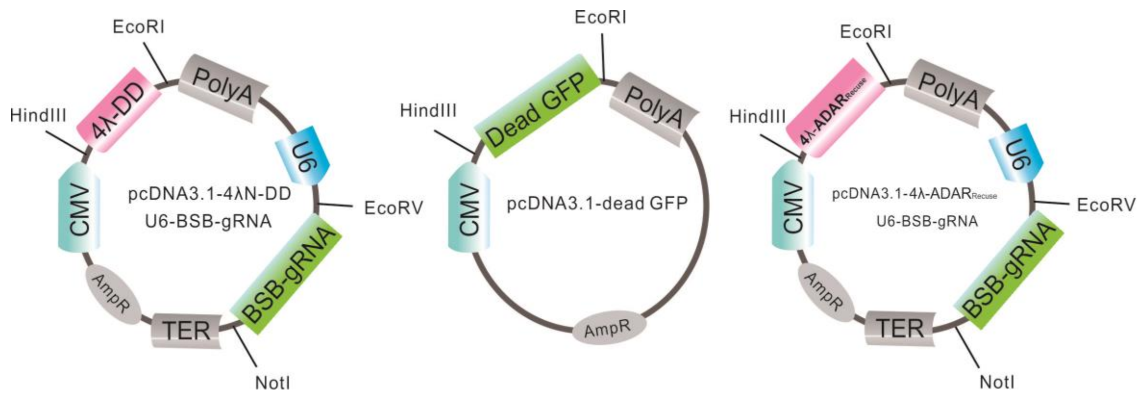

Figure S8. **The Plasmid maps used in the work.** The E. coli strains used in this study were Trelief™ 5α Chemically Competent Cells, purchased from Tsingke Biotechnology (Beijing, China). The 4λN-DD (E488Q), 4λN-ADAR$_{RESCUE}$, and Dead GFP reporter genes were cloned into a modified pcDNA3.1 zeo (+)-U6 vector at the Hind-III and Eco-RI restriction endonuclease sites. For the investigated guide RNA, it was cloned into a modified pcDNA3.1 zeo (+) vector at the Eco-RV and Not-I restriction endonuclease sites.

References

1. Montiel-Gonzalez, MF, Vallecillo-Viejo, IC, and Rosenthal, JJC (2016). An efficient system for selectively altering genetic information within mRNAs. *Nucleic Acids Research* **44**.
2. Abudayyeh, OO, Gootenberg, JS, Franklin, B, Koob, J, Kellner, MJ, Ladha, A, *et al.* (2019). A cytosine deaminase for programmable single-base RNA editing. *Science* **365**: 382-+.
