# Supplementary material for: Enhancing RNA editing efficiency and specificity with engineered ADAR2 guide RNAs
Source: Mol Ther Nucleic Acids. 2025 Jan 13;36(1):102447. doi: 10.1016/j.omtn.2025.102447 (PMC11834095; doi:10.1016/j.omtn.2025.102447)
Supplement: Document S2. Article plus supplemental information [file mmc2.pdf]

Molecular Therapy
Nucleic Acids
Original Article

Enhancing RNA editing efficiency and specificity with engineered ADAR2 guide RNAs

Xilei Ai,[1,2] Sheng Ding,[3] Shan Zhou,[1] Feng Du,[1] Shuai Liu,[1] Xin Cui,[1] Juan Dong,[1] Xin Huang,[1] and Zhuo Tang[1]

[1]Natural Products Research Center, Chengdu Institute of Biology, Chinese Academy of Sciences, Chengdu 610041, China; [2]Academy of Chinese Medical Sciences, Henan University of Chinese Medicine, Zhengzhou 450046, China; [3]School of Clinical Medical College & Affiliated Hospital, Chengdu University, Chengdu 610052, China

RNA editing is a prospective therapeutic approach for correcting harmful mutations, offering the benefits of reversibility and tunability without permanently modifying the genome. However, the relatively low enzymatic activity and the occurrence of off-target editing events present significant challenges, limiting its utility. In response to this limitation, we introduced a novel strategy: strand displacement-responsive ADAR system for RNA editing (SPRING) by adding a "blocking sequence" to form a hairpin guide RNA. This modification significantly improves the efficiency of site-directed RNA editing (SDRE) at various target sites. Furthermore, the use of hairpin guide RNA within the SPRING system enhances the specificity of RNA editing through competitive reactions during target hybridization. In principle, this approach can be employed across various ADAR-based editing systems, offering a novel RNA-editing platform with wide-ranging potential for research, therapy, and biotech applications.

INTRODUCTION

Single nucleotide polymorphisms (SNPs) are the primary form of genetic variation observed in individuals, and they are particularly prevalent. Point mutations, which are specific types of SNPs, contribute significantly to the genetic variations associated with diseases, making up approximately half of all such variations. Transition mutations in particular contribute to 60% of these harmful point mutations.[1,2] As a result, the development of novel gene editing methods to address these mutations has progressed rapidly. Base editors and primer editors, which are derived from CRISPR-Cas9, have emerged as powerful tools for addressing point mutations in DNA without the need for double-strand breaks. These innovative techniques are extensively utilized in both research and the treatment of genetic disorders, showcasing their effectiveness in the fields of genetic disease studies and therapies.[1,3,4] However, base editors induce permanent DNA alterations and are burdened by issues such as off-target editing, immune responses, and other limitations.[5–8] These limitations constrain their potential applications in the field of genetic disease therapy. In contrast, RNA base editors exclusively modify RNA bases and do not introduce permanent changes to DNA, offering unique advantages for therapeutic applications.[9]

Site-directed RNA editing (SDRE) is a precision strategy for altering a specific RNA base within given mRNAs. By attaching the catalytic domain of the RNA-editing enzyme ADAR to an antisense guide RNA, targeted adenosines can undergo conversion into inosines. Notably, the edited base is identified as guanosine by the translation and splicing machinery due to the structural similarity between inosine and guanosine. Endogenous ADAR-based methods such as RESTORE,[10] LEAPER,[11] CLUSTER,[12] and others[13] have shown the research and therapeutic promise of RNA A-to-I base editing. However, endogenous ADAR's efficiency in RNA editing is relatively low in the majority of cells. Additionally, its use is restricted to editing adenosines within particular RNA motifs preferred by native endogenous ADARs, and the effectiveness of this editing procedure may be impacted by their variable expression levels in different tissues.[14] Furthermore, it cannot be employed for innovative functions like the conversion of cytosine to uracil (C to U), which necessitates the introduction of exogenous ADAR2 variants.[15]

By utilizing exogenous editors to free the editing process from dependence on native ADARs, Rosenthal and colleagues employed a guide RNA (gRNA) with a BoxB aptamer capable of specifically binding to the λ phage N protein (λN) fused with ADAR.[16] This strategy is known as the BoxB-λN-ADAR system. Through Watson-Crick base pairing, the gRNA can create a duplex with the target mRNA. The target adenosine (A) at the C/A mismatch site would then undergo hydrolytic deamination by ADAR proteins, converting it to inosine (I). Furthermore, noncovalent recruitment of exogenous ADAR by other fusion proteins, like MCP-ADAR,[17–19] Cas13-ADAR,[20] and Cas9-ADAR,[21] has been developed to enhance editing efficiency. In the MS2-MCP-ADAR approach, MCP was fused with ADAR and bound to the target RNA using a gRNA that contained an RNA aptamer (MS2) of MCP, much like the BoxB-λN-ADAR

Received 28 October 2023; accepted 10 January 2025;
https://doi.org/10.1016/j.omtn.2025.102447.

Correspondence: Xin Huang, Natural Products Research Center, Chengdu Institute of Biology, Chinese Academy of Sciences, Chengdu 610041, China.
E-mail: huangxin@cib.ac.cn
Correspondence: Zhuo Tang, Natural Products Research Center, Chengdu Institute of Biology, Chinese Academy of Science, Chengdu 610041, China.
E-mail: tangzhuo@cib.ac.cn

Published by Elsevier Inc. on behalf of The American Society of Gene and Cell Therapy.
This is an open access article under the CC BY license (http://creativecommons.org/licenses/by/4.0/).

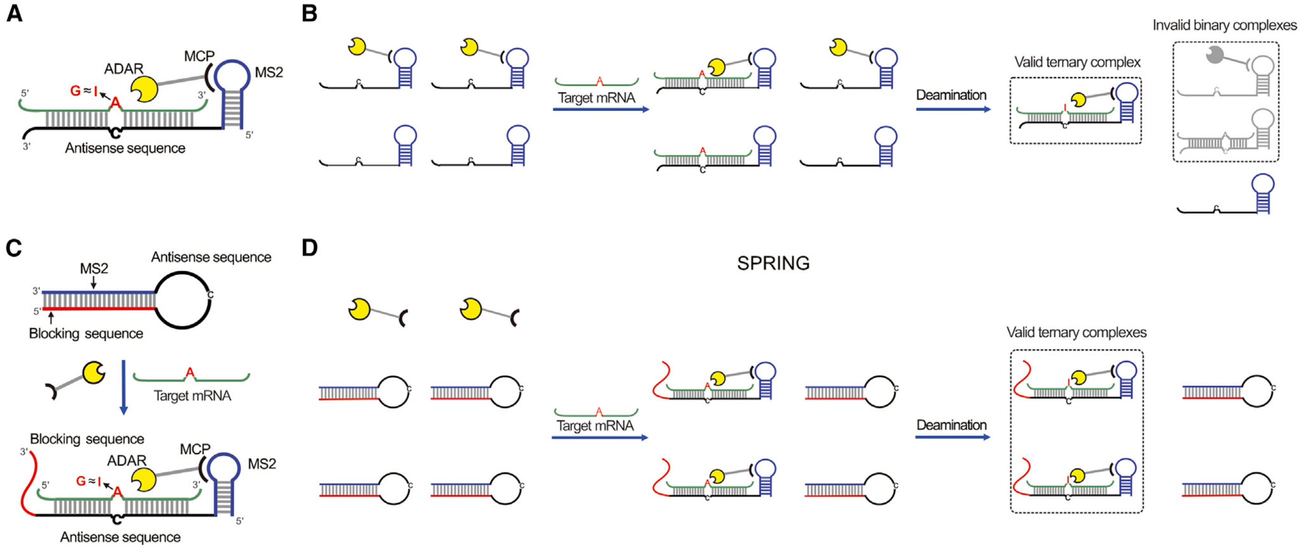

Figure 1. SPRING strategy for enhanced RNA editing efficiency

(A) Schematic of the MS2-MCP-ADAR system-mediated A-to-G editing. (B) Schematic of the MS2-MCP-ADAR system for SDRE; the free-state gRNA without the blocking sequence will compete with binding gRNA for ADAR. (C) Schematic of the MS2-gRNA with blocker-mediated A-to-G editing. (D) ADAR is recruited only by gRNA with the blocking sequence bound to the substrate.

system. To specifically identify RNA targets, Cas13 and Cas9 fused with ADAR rely on the corresponding gRNAs. Additionally, SNAP-tagged ADAR and chemically altered gRNA with benzyl guanine are used to covalently link the exogenous ADAR enzyme and gRNA in order to increase editing yields *in cellula*.[22–26] However, the fusion systems have faced challenges due to their relatively low enzymatic activity and the risk of off-target effects, which have limited their applications as RNA editing tools. A potential solution to overcome these limitations is to screen for variants within a mutagenesis pool using phenotypic selection methods based on cell fitness or fluorescent reporters.[27,28] Nevertheless, the discovery of effective ADAR variants through this approach has proven to be highly limited and extremely challenging. Only one variant, ADARE488Q, was successfully selected to increase enzymatic activity and is now widely utilized for biotechnological and therapeutic applications.[28] There is a clear need for alternative methods to boost editing efficiency.

Given these challenges, we hypothesized that a significant limitation of existing RNA editing systems is the overabundance of gRNA, which can lead to non-functional gRNA/mRNA complexes. These complexes could reduce editing efficiency and increase the potential for off-target effects. To address this, we proposed the incorporation of a blocking sequence into the gRNA to modulate its function. By extending the gRNA with a blocking sequence complementary to the gRNA aptamer, we aimed to prevent the formation of these non-functional binary complexes and enhance the specificity and efficiency of RNA editing. In this study, we introduce a novel strategy called the strand displacement-responsive ADAR system for RNA editing (SPRING), which leverages this blocking sequence design to address the limitations of current RNA editing methods.

RESULTS

Design and optimization of BSM-gRNA

As shown in Figure 1A, the prototypical MS2-MCP-ADAR system comprises an ADAR-MCP fusion protein and an MS2-gRNA. The MS2 gRNA consists of an MS2 aptamer (blue), which could bind to the fusion protein specifically, and an antisense sequence (black). This configuration not only enables the formation of a duplex with the target mRNA through Watson-Crick base pairing but also plays a crucial role in recruiting the ADAR enzyme to the target site.[29] The MCP protein is fused to the deaminase domain (DD) of the human ADAR2 mutant (E488Q). This specific mutation (E488Q) in ADAR2 has been known to enhance the enzyme's activity and affinity.[28] The ADAR proteins hydrolytically deaminate the target A at the C-A mismatch site, converting it into I, which can be interpreted as guanosine (G) by RNA splicing and translation machinery. Given that the targeted RNA editing process relies on the formation of the mRNA/ADAR-MCP/MS2-gRNA ternary complex, we speculate that the presence of binary complexes (mRNA/MS2-gRNA or ADAR-MCP/MS2-gRNA) would certainly influence the efficiency of RNA base editing (as illustrated in Figure 1B). To effectively eliminate the impact of those invalid binary complexes on the deamination process, we developed the SPRING strategy, designed to maximize the formation of the desired valid ternary complexes in the presence of target mRNA, significantly enhancing targeted RNA editing efficiency. As shown in Figure 1C, the blocking sequence (red), which was integrated at the 5′ end of MS2-gRNA (thus creating BSM-gRNA), was deliberately designed to be complementary to the sequence of MS2, forming a hairpin structure. Hence, the BSM-gRNA remains in the off state when mRNA is absent, as MS2 is enclosed within the hairpin structure; therefore, it will not bind with

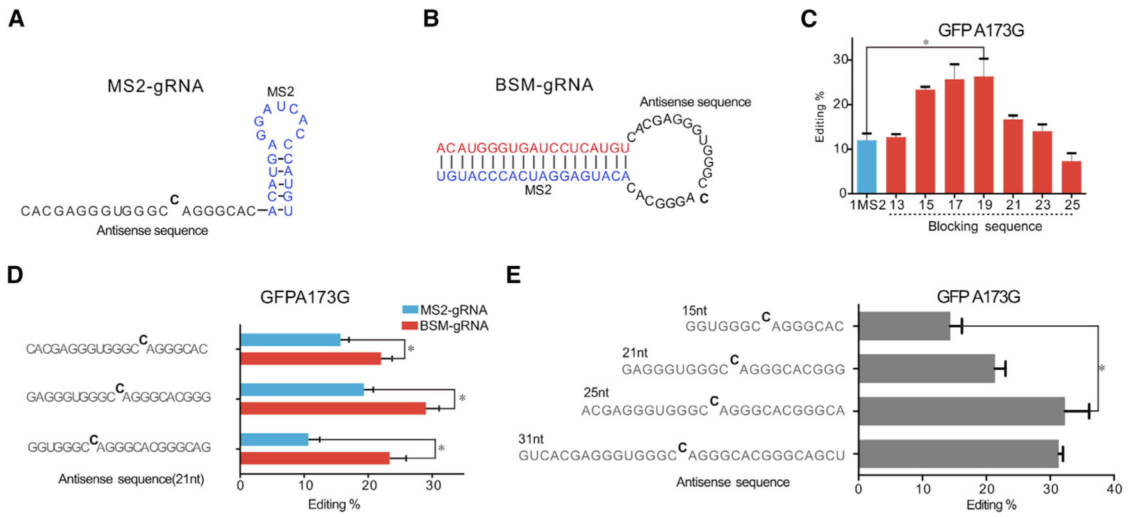

Figure 2. BSM-gRNA mediated RNA editing

(A) Schematic of the MS2-gRNA. (B) Schematic of the BSM-gRNA containing a blocking sequence. (C) ADAR-mediated editing efficiencies with the addition of plasmids expressing MS2-gRNA and BSM-gRNA that target the same site (deadGFP). The length of the blocking sequence varied from 13 to 25 nt. All gRNA is expressed from a U6 promoter. (D) Optimization of BSM-gRNA. Distances from the ADAR2 recruiting region were systematically varied. (E) Optimization of BSM-gRNA. Lengths from the ADAR2 recruiting region were systematically varied. All values are mean ± SEM with 3 biological replicates. Student's t test: $^{*}p < 0.05$, $^{**}p < 0.01$, $^{***}p < 0.001$.

ADAR-MCP. However, when target mRNA is present, BSM-gRNA can recognize the mRNA by forming an RNA duplex, subsequently releasing MS2 to facilitate the recruitment of ADAR-MCP at the editing site (Figure 1D). A similar strand replacement strategy has been successfully applied in various areas, including CRISPR regulation, cell organelle imaging, and transcriptional control, implying the feasibility of our approach.[30–34] Therefore, we hypothesized that this approach could significantly diminish the formation of invalid binary complexes and enhance the likelihood of the editing system engaging in target deamination.

To detect editing activities in the SPRING system, we employed a non-functional GFP variant (W58X) with a premature stop codon (UAG) resulting from a G173A mutation. This specific mutation can be corrected through ADAR-mediated A-to-I RNA editing (Figure 2A), and we assessed the system's performance by conducting RT-PCR/Sanger sequencing. As the crucial element of the SPRING strategy, the design of BSM-gRNA (Figure 2B) must meet two key criteria. (1) In the absence of a substrate, an adequate length of the blocking sequence should be present to facilitate the formation of a double-stranded hairpin structure. This structure serves to disrupt the interaction between MS2 and MCP-ADAR. (2) In the presence of the target mRNA, a change in the hairpin structure should be triggered, allowing the MS2 aptamer to revert to its original configuration, thereby activating the RNA editing system. Therefore, we designed seven BSM-gRNAs with blocking sequences of different lengths, ranging from 13 to 25 nt (Figure S1). To assess their editing efficiency, we subsequently created vectors that included these modified gRNAs, along with GFP (W58X) and MCP-DD (E488Q) fusion proteins, respectively. All of these constructs underwent confirmation through DNA sequencing before being transfected into human embryonic kidney cells (HEK293T). As shown in Figure 2C, in comparison to the original MS2-gRNA, the RNA editing efficiency of BMS-gRNA gradually increased as the blocker length extended from 13 to 19 nt. This demonstrates the effectiveness of the SPRING strategy in enhancing the editing efficiency of the MS2-MCP-ADAR system. Conversely, RNA editing efficiency gradually decreased when the blocker lengths exceeded 19 nt, indicating that excessive blocker lengths hinder strand displacement. Notably, the original MS2-MCP-ADAR system exhibited only 12% editing at the dead GFP A173 site, while the newly designed SPRING system with a 19-nt blocking sequence displayed approximately 2.2-fold higher efficiency (26% editing) than the original system. Subsequently, we examined the influence of target A's position on the editing efficiency of the SPRING system. The results indicated that superior editing efficiency was achieved when target A was positioned in the middle of the targeted sequence (Figure 2D). Additionally, we optimized the length of the antisense sequence and found that a 25-nt antisense sequence improved editing efficiency by 32% (Figure 2E). Consequently, we selected a 19-nt blocking sequence and a 25-nt antisense sequence with the target position in the middle for subsequent experiments.

Using the BoxB-λN-ADAR system improves editing efficiency

To further enhance RNA editing efficiency, we replaced the MS2 aptamer in the MS2-MCR-ADAR system with a smaller BoxB aptamer from the BoxB-λN-ADAR system, which is also commonly used.[16,35–37] Additionally, it has been established that four λNs linked to the DD (E488Q) result in higher editing efficiency.[16] Upon adding the blocking sequence to the 5′-end of BoxB-gRNA, the editing efficiency of BSB-gRNA increased by 1.4-fold compared to

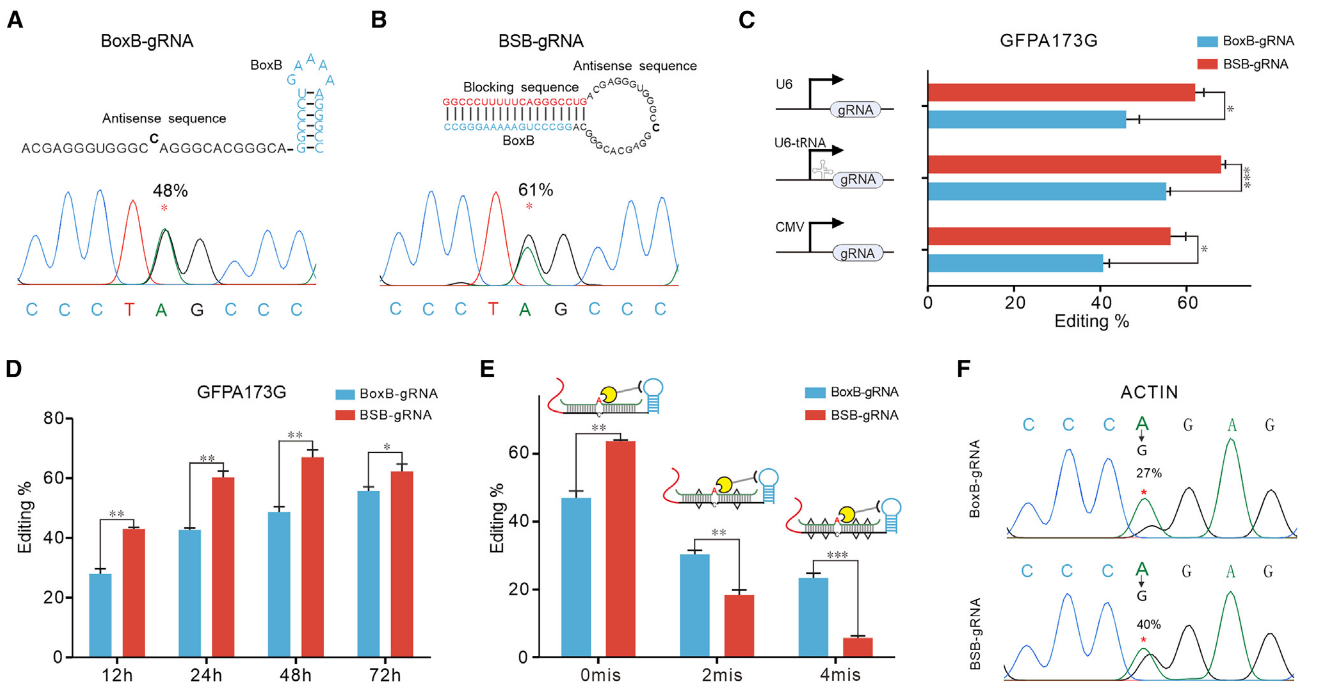

Figure 3. BSB-gRNA mediated RNA editing

(A) Schematic of the BoxB-gRNA (top) and sequence chromatograms of cDNA from edited dGFP-W58X with BoxB-gRNA (bottom). (B) Schematic of the BSB-gRNA containing a blocking sequence (top) and sequence chromatograms of cDNA from edited dGFP-W58X with BSB-gRNA (bottom). (C) RNA editing efficiencies were achieved 48 h post transfection of various promoter designs. All values are mean ± SEM with 3 biological replicates. Statistical analysis was done using a two-tailed Student's t test: $^*p < 0.05$, $^{**}p < 0.01$, $^{***}p < 0.001$. (D) The ORF GFP 173A was edited to measure the time course for 3 days. The RNA editing percentage was then directly measured by RT-PCR-Sanger sequencing. (E) Schematic of the BoxB-gRNA and BSB-gRNA with mismatches. After 48 h of induction, RNA levels were measured by RT-PCR-Sanger sequencing after the editing biosensor had been incubated for 24 h following transfection. (F) Sequence chromatograms of cDNA from edited ACTIN-CAG edited with BSB-gRNA and BoxB-gRNA.

BoxB-gRNA (as illustrated in Figures 3A, 3B and S2). This result indicates that the SPRING strategy remains effective in the context of the BoxB-λN-ADAR system. Furthermore, when compared with the BSM-MCP-ADAR system, the BSB-λN-ADAR system enhanced the editing efficiency to 61% at the dead GFP 173A site. We proceeded to investigate the influence of various transcript promoters, including U6, CMV, and U6-tRNA promoters, on RNA editing using the SPRING system. CMV promoters have been effectively utilized previously to enhance shRNA synthesis, leading to improved gene silencing capabilities.[38] Additionally, the U6-tRNA promoter, as reported by Yang's group, has been shown to enhance the expression of gRNAs for Cas9-based gene editing, with processing by RNase P and RNase Z contributing to the generation of accurate and clean RNA tools.[39] The results indicate that the U6-tRNA promoter can improve editing efficiency, reaching 68% compared to the 62% achieved with the original U6 promoter. Statistical analysis showed that this improvement was significant in the BoxB-gRNA group (p = 0.0426) but not in the BSB-gRNA group (p = 0.0550) (Figures 3C and S3). The 4λN-ADAR(E488Q) expression driven by the stronger CMV promoter exhibited superior editing efficiency compared with SV40 (Figure S4). Additionally, when we introduced 4λNs-DD into the cells, targeting the GFP 173A site, we monitored the editing efficiency over time through RT-PCR/Sanger sequencing. It became apparent that the editing peak occurred 48 h after transfection with the optimized SPRING system, as depicted in Figure 3D. Furthermore, we conducted a comparative analysis of RNA editing efficiency using our system in comparison with the commonly used 2BoxB-gRNA system, which contains two BoxB aptamers at both termini of the antisense part. As shown in Figure S5, BSB-gRNA exhibited significantly higher editing efficiency compared to BoxB-gRNA, but there was no significant difference between BSB-gRNA and 2BoxB-gRNA (p = 0.2473, NS).

The addition of a blocking sequence into the gRNA has greatly enhanced the efficiency of ADAR-based RNA editing. In light of this enhancement, our attention has shifted toward evaluating the specificity of the SPRING system. Mali and colleagues enhanced specificity by splitting the DD into two catalytically inactive fragments that merge to create a catalytically active enzyme exclusively at the intended target.[40] Nevertheless, this split protein approach led to a decrease in off-target editing while simultaneously reducing on-target editing efficiency.[40] Katrekar et al. successfully decreased off-target editing while preserving on-target editing efficiency through the relocation of the editing enzymes from the cytoplasm to the nucleus.[17]

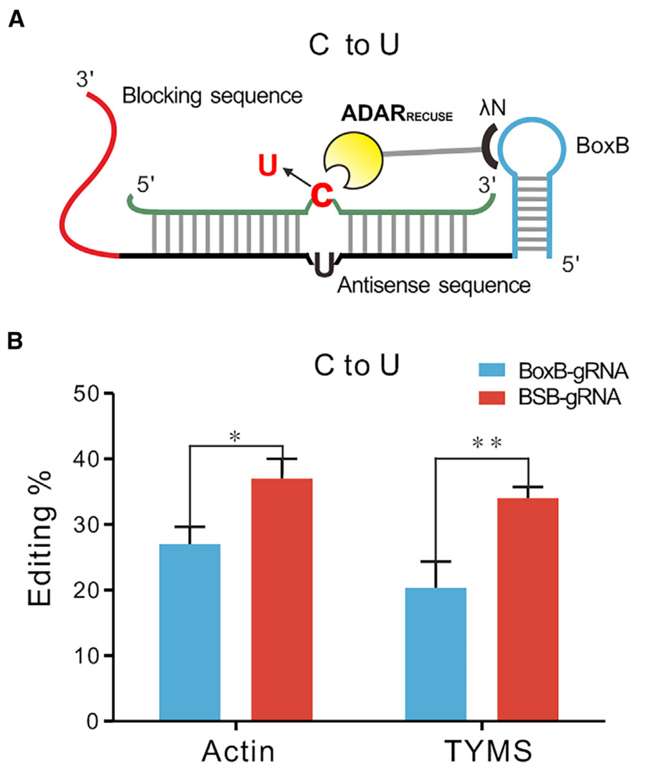

Figure 4. Using the strategy-mediated C-to-U editing

(A) Schematic of the BSB-λN- $ADAR_{RESCUE}$-mediated C-to-U editing. (B) $ADAR_{RESCUE}$ contains mutations of ADAR2 (E488Q/V351G/S486A/T375S/S370C/P462A/N597I/L332I/I398V/K350I/M383L/D619G/S582T/V440I/S495N/K418E).[15] λN-$ADAR_{RESCUE}$ edited the sites in the ACTIN and TYMS ORF with BoxB-gRNA or BSB-gRNA in HEK293T cells. After 48 h of induction, RNA levels were measured by RT-PCR-Sanger sequencing after the editing biosensor had been incubated for 24 h following transfection. All values are mean ± SEM with 3 biological replicates. Student's t test: $^{*}p < 0.05$, $^{**}p < 0.01$, $^{***}p < 0.001$.

Nevertheless, when applied in the MS2-MCP-ADAR system, it has demonstrated ineffectiveness in decreasing off-target effects; instead, it compromises on-target editing.[17] As a result of the stable stem-loop structure in BSB-gRNA, the binding process with the target RNA requires the opening of this structure. Consequently, it demands more precise pairing and fewer mismatches between the gRNA and the targeted sequence. To test this hypothesis, we introduced non-complementary bases into the antisense sequences of BoxB-gRNA and BSB-gRNA to create mismatches when they hybridized with mRNA, thus simulating their potential binding to non-target strands. As illustrated in Figure 3E, the editing efficiency of BoxB-gRNA at the 173A site of the dead GFP decreased from 47% to 30% with two mismatches and further dropped to 23% with four mismatches. For BSB-gRNA, the editing efficiency decreased from 64% to 18% with two mismatches and dramatically decreased to 6% with four mismatches, indicating a significant reduction in RNA editing in the presence of mismatches. Next, we sought to evaluate the specificity of BSB-gRNAs at both the transcriptome-wide and target transcript levels. To assess the former, we performed deep RNA sequencing (RNA-seq) analysis on BoxB-gRNA and BSB-gRNA samples and an untransfected HEK293FT sample. Notably, compared to BoxB-gRNA, which exhibited nearly 30,000 off-target sites, BSB-gRNA showed a substantial reduction in off-target editing, with approximately 12,000 off targets observed (Figure S6). This underscores the efficiency and specificity of the SPRING system, surpassing the performance of the original BoxB-λN-ADAR system. After confirming the efficacy of the SPRING strategy in editing exogenous mRNA, we evaluated its performance in endogenous transcripts. 176A within the open reading frame (ORF) of ACTIN was selected as the editing target,[10] a site that has been widely used by other RNA-based editor systems. Compared to original BoxB-gRNA, the A-to-I RNA editing efficiency of the target increased from 27% to 40% based on BSB-gRNA (Figures 3F and S7), signifying the most substantial improvement in RNA editing observed at this location.

Using the strategy in the $ADAR_{RESCUE}$ system

The ADAR-based RNA editing system is exclusively effective for A-to-I editing. Zhang's group successfully transformed the human ADAR2 DD into a bifunctional enzyme ($ADAR_{RESCUE}$) through protein evolution, allowing it to deaminate not only A but also cytidine.[15] Consequently, we proceeded to assess whether the SPRING strategy remained effective in another C-to-U system, thus expanding the range of treatable disease mutations and protein modifications. We substituted the ADAR2 mutant (E488Q) with $ADAR_{RESCUE}$ within the optimized BoxB-λN-ADAR system (Figure 4A; Table S3) and selected two TCG editing targets within the ORF of endogenous transcripts, ACTIN and TYMS, respectively. As shown in Figure 4B, a remarkable increase in the editing levels of the two targeted transcripts edited by BSB-gRNAs was observed when compared to BoxB-gRNA. Specifically, at the ACTIN gene editing site, we observed an increase in C-to-U editing efficiency from 27% to 37%. At the TYMS editing locus, the editing efficiency increased from 20% to 34%. In conclusion, these results confirm that our strategy remains effective in C-to-U RNA editing.

DISCUSSION

Without modifying the genome, SDRE facilitated by ADARs enables precise gene editing by converting A to I. In contrast to CRISPR-based gene editing techniques, RNA editing events are transient, reducing the risk of long-lasting unintended side effects. This mitigates concerns associated with off-target edits compared to DNA-targeting methods. Additionally, compared to RNA editing tools based on Cas13 protein, such as REPAIR and REPAIRx,[41] the CRISPR-free ribonucleoprotein-based systems, including the self-labeling SNAP tag and λN-BoxB system as well as the MS2-MCP system, can avoid inducing an immune response.[13] Its compact size also aids in its integration into gene therapy vectors, such as adeno-associated virus vectors (AAVs), which have limited additional gene space. Moreover, RNA editing methods utilizing endogenous ADARs have been reported to mitigate issues associated with the exogenous expression of engineered ADAR enzymes. However, the primary drawback of these methods lies in the inconsistent editing efficiency due to the varying expression levels of endogenous ADAR

proteins across different cell lines.[13] RNA editing tools based on engineered ADAR fusion are independent of endogenous ADAR levels within host cells, ensuring stable editing efficiency. Nevertheless, it remains crucial to minimize off-target editing resulting from robust ADAR activity. To improve RNA editing efficiency, we developed the SPRING strategy by modifying gRNA, which distinguishes itself from the traditional approach of directly evolving ADAR proteins. A hairpin gRNA can be activated by the target mRNA, facilitating the creation of a functional RNA editing complex. This strategy exhibits several advantages. The efficiency of RNA editing was enhanced by maximizing the formation of valid ternary complexes and reducing the occurrence of invalid binary complexes. The design of the hairpin gRNA is simple, and it is easy to operate and exhibits a high degree of generality. In theory, this strategy can be applied to various ADAR-based editing systems, including REPAIR,[20] RESTORE,[10] and CIRTS.[3] This strategy effectively eliminates off-target editing caused by gRNA mismatches in editing tools, but it does not reduce off-target editing caused by effectors. The SPRING system is not only suitable for A-to-I RNA editing but also for C-to-U RNA editing. Using the optimized SPRING system, we attained an editing efficiency of 67% at the exogenous deadGFP 173A site along with increased specificity. Notably, we observed substantial improvements in A-to-I and C-to-U RNA editing for endogenous targets. We believe that combining this strategy with existing methods capable of reducing off-target effects induced by effectors, such as splitting ADAR deaminases[40] or incorporating nuclear localization signals,[37] can further enhance the specificity of RNA editing. In conclusion, SPRING offers a novel RNA editing platform with wide-ranging potential for research, therapy, and biotech applications.

MATERIALS AND METHODS

Reagents

HEK293T cells obtained from the Cell Bank of the Type Culture Collection of the Chinese Academy of Sciences were used in this study. The cell culture utilized a low-glucose version of Gibco's Dulbecco's modified Eagle's medium (DMEM), supplemented with Gibco fetal bovine serum and Beyotime antibiotics, including penicillin and streptomycin. Milli-Q water (18 MXcm, Millipore), trypsin-EDTA (0.25%), and 10× PBS buffer (Sangon Biotech) were used for cell culture processes. Hieff Trans liposomal transfection reagent (Yeasen) was used for transfection. Other reagents, such as magnesium chloride hexahydrate from Sinopharm Chemical Reagent, L-glutamine (Gibco), and potassium chloride from Sinopharm Chemical Reagent, were purchased from Sigma-Aldrich, Thermo Fisher Scientific, or New England Biolabs unless otherwise specified.

Plasmid constructions

The *E. coli* strains used in this study were Trelief 5α chemically competent cells, purchased from Tsingke Biotechnology (Beijing, China). The MS2-MCP plasmids were obtained from Prof. Shaohua Yao of Sichuan University and subsequently modified for use in our experiments. The investigated gRNA was cloned into a modified pcDNA3.1 zeo (+) vector at the Eco-RV and Not-I restriction endonuclease sites (Table S1; Figure S8). The DNA sequences of the investigated genes were amplified using primers and purified using a PCR clean kit. The purified DNA fragments and pcDNA3.1 zeo (+)-U6 vector were then subjected to overnight digestion at 37°C with Eco-RV (New England Biolabs [NEB], USA) and Not-I (NEB). The DNA fragments and vectors were digested and recovered using the E.Z.N.A. Gel Extraction Kit. Then, T4 ligase was used to ligate the fragments at 4°C overnight to form the ligation products. Chemically competent cells were transformed with the ligation products (Vazyme). Finally, a DNA sequencing analysis was performed to confirm the presence of the inserted sequences on the plasmid. The 4λN-DD (E488Q), 4λN-$ADAR_{RESCUE}$, and dead GFP reporter genes were cloned into a modified pcDNA3.1 zeo (+)-U6 vector at the Hind-III and Eco-RI restriction endonuclease sites (Table S2; Figure S8). The DNA of the genes of interest was amplified using Fidelity DNA polymerase (Vazyme) and purified with PCR clean kits. The purified samples were then subjected to overnight digestion at 37°C, where the purified DNA fragments and pcDNA3.1(+) vector were digested with Hind-III (NEB). Subsequently, the purified inserts and vector DNA were ligated using T4 ligase (Vazyme) following the manufacturer's instructions.

Mammalian cell culture and transfection

Mammalian cell culture

We cultured HEK293T cells in DMEM (Gibco) supplemented with 200 units/mL penicillin, 200 units/mL streptomycin sulfate, and 2 mM L-glutamine. The medium also contained 10% (v/v) fetal bovine serum (Invitrogen). The cells were maintained at 37°C in a humidified 5% CO_2 incubator.

Transfection

HEK293 cells were cultured in 24-well plates and transfected at approximately 70%–80% confluence with either 500 ng of pcDNA3.1-4λN-DD-gRNA (the plasmid targeting dead GFP173A) and 500 ng of pcDNA3.1-deadGFP reporter or 1,000 ng of pcDNA3.1-4λN-DD-gRNA (the plasmid targeting endogenous genes). Transfections were performed using Hieff Trans liposomal transfection reagent (Yeasen) following the manufacturer's instructions.

Editing efficiency quantification

In our experiments, transfected HEK293T cells were utilized to quantify the efficiency of editing in mammalian cells. For RNA editing experiments involving reporter genes in cell culture, we used 500 ng of the reporter plasmid followed by 500 ng of the gRNA/enzyme plasmid. For *in cellula* RNA editing experiments targeting either endogenous transcripts or disease-simulated reporters, 1,000 ng of the gRNA/enzyme plasmid was used. All experiments were conducted in 24-well plates. The cells were transfected when they reached 80% confluence. After 24 h, media were exchanged, and the cells were incubated for an additional 48 h (for endogenous/disease targets, media were changed every 24 h) before further processing.

To isolate cells from a sample, we extracted total RNA using the Cell Total RNA Isolation Kit V2 (Vazyme). Following RNA purification,

the obtained RNA was reverse transcribed using an oligo-dT and random hexamer primers, following the manufacturer's instructions, instead of the First-Strand cDNA Synthesis Kit (Vazyme). The resulting cDNA product was then PCR amplified using target-specific primers (listed in Table S3) and sent to Tsingke (Beijing, China) for analysis using Fidelity DNA polymerase (Vazyme). The peak heights of the bases A and G, as measured by Software Scanner 1.0 (Applied Biosystems), were used to calculate the editing ratio at each site using the formula A / [A + G].

Transcriptome-wide RNA-seq analysis

Plasmids expressing either BoxB-gRNA-GAPDH or BSB-gRNA-GAPDH were transfected into HEK293T cells. Cells were collected 48 h after transfection, and total RNA was purified using the Zymo Research Direct-zol RNA Miniprep Kit (catalog number R2051). High-quality mRNA was isolated using the NEBNext Poly(A) mRNA Magnetic Isolation Module (NEB, E7490L). The isolated mRNA was randomly fragmented into a size range of 300–350 nt using an Mg^{2+} buffer. Library preparation was then performed using the NEBNext Ultra II RNA Library Prep Kit (NEB, E7770L). Paired-end 150 sequencing was conducted on the Illumina NovaSeq 6000 system, generating 6 Gb of data per sample. Transcriptome data were quality controlled and adapter trimmed using Trimmomatic, and the sequences were mapped to the reference genome using Hisat2. BAM files were down-sampled to the same sequence count using Samtools. SNP calling was performed using GATK, with filtering criteria set to remove variants with MQ < 20, DP < 20, and QUAL < 13. Given that NGS sequencing is bidirectional, A-to-G and T-to-C mutations were extracted from the sequencing results. For mutations present in both the sample and control groups, Fisher's exact test was applied. Mutations with $p < 0.01$ and a mutation count in the sample group greater than 1.1 times that in the control group were retained. Mutations present only in the sample group and absent from the control group were directly retained.

DATA AND CODE AVAILABILITY

The data that support the findings of this study are available from the corresponding author upon reasonable request.

ACKNOWLEDGMENTS

This research was funded by the Sichuan Science and Technology Program (2023NSFSC0016, 2022YFS0012, and 2022YFS0432), the National Natural Science Foundation of China (22177111 and 22377124), the Central Guidance on Local Science and Technology Development Fund of Sichuan Province (2022ZYD0090), and the Key Projects of Chengdu Institute of Biology, Chinese Academy of Sciences (CIBGG202303).

AUTHOR CONTRIBUTIONS

Conceptualization, Z.T.; methodology, Z.T.; investigation, X.A., S.D., and S.Z.; formal analysis, X.A., S.D., and S.L.; writing – original draft, X.A.; writing – review & editing, Z.T. and X.H.; funding acquisition, Z.T.; resources, X.C., F.D., and J.D.; supervision, Z.T.

DECLARATION OF INTERESTS

The authors declare no competing interests.

SUPPLEMENTAL INFORMATION

Supplemental information can be found online at https://doi.org/10.1016/j.omtn.2025.102447.

REFERENCES

1. Gaudelli, N.M., Komor, A.C., Rees, H.A., Packer, M.S., Badran, A.H., Bryson, D.I., and Liu, D.R. (2017). Programmable base editing of A.T to G.C in genomic DNA without DNA cleavage. Nature *551*, 464–471.
2. Landrum, M.J., Lee, J.M., Benson, M., Brown, G., Chao, C., Chitipiralla, S., Gu, B., Hart, J., Hoffman, D., Hoover, J., et al. (2016). ClinVar: public archive of interpretations of clinically relevant variants. Nucleic Acids Res. *44*, D862–D868.
3. Rauch, S., He, E., Srienc, M., Zhou, H., Zhang, Z., and Dickinson, B.C. (2019). Programmable RNA-Guided RNA Effector Proteins Built from Human Parts. Cell *178*, 122–134.e12.
4. Anzalone, A.V., Randolph, P.B., Davis, J.R., Sousa, A.A., Koblan, L.W., Levy, J.M., Chen, P.J., Wilson, C., Newby, G.A., Raguram, A., and Liu, D.R. (2019). Search-and-replace genome editing without double-strand breaks or donor DNA. Nature *576*, 149–157.
5. Park, S., and Beal, P.A. (2019). Off-Target Editing by CRISPR-Guided DNA Base Editors. Biochemistry *58*, 3727–3734.
6. Simeonov, D.R., Brandt, A.J., Chan, A.Y., Cortez, J.T., Li, Z., Woo, J.M., Lee, Y., Carvalho, C.M.B., Indart, A.C., Roth, T.L., et al. (2019). A large CRISPR-induced bystander mutation causes immune dysregulation. Commun. Biol. *2*, 70.
7. Zhou, C., Sun, Y., Yan, R., Liu, Y., Zuo, E., Gu, C., Han, L., Wei, Y., Hu, X., Zeng, R., et al. (2019). Off-target RNA mutation induced by DNA base editing and its elimination by mutagenesis. Nature *571*, 275–278.
8. Zuo, E., Sun, Y., Wei, W., Yuan, T., Ying, W., Sun, H., Yuan, L., Steinmetz, L.M., Li, Y., and Yang, H. (2019). Cytosine base editor generates substantial off-target single-nucleotide variants in mouse embryos. Science *364*, 289–292.
9. Booth, B.J., Nourreddine, S., Katrekar, D., Savva, Y., Bose, D., Long, T.J., Huss, D.J., and Mali, P. (2023). RNA editing: Expanding the potential of RNA therapeutics. Mol. Ther. *31*, 1533–1549.
10. Merkle, T., Merz, S., Reautschnig, P., Blaha, A., Li, Q., Vogel, P., Wettengel, J., Li, J.B., and Stafforst, T. (2019). Precise RNA editing by recruiting endogenous ADARs with antisense oligonucleotides. Nat. Biotechnol. *37*, 133–138.
11. Qu, L., Yi, Z., Zhu, S., Wang, C., Cao, Z., Zhou, Z., Yuan, P., Yu, Y., Tian, F., Liu, Z., et al. (2019). Programmable RNA editing by recruiting endogenous ADAR using engineered RNAs (vol 37, pg 1059, 2019). Nat. Biotechnol. *37*, 1380.
12. Reautschnig, P., Wahn, N., Wettengel, J., Schulz, A.E., Latifi, N., Vogel, P., Kang, T.-W., Pfeiffer, L.S., Zarges, C., Naumann, U., et al. (2022). CLUSTER guide RNAs enable precise and efficient RNA editing with endogenous ADAR enzymes in vivo. Nat. Biotechnol. *40*, 759–768.
13. Song, J., Zhuang, Y., and Yi, C. (2024). Programmable RNA base editing via targeted modifications. Nat. Chem. Biol. *20*, 277–290.
14. Uzonyi, A., Nir, R., Shliefer, O., Stern-Ginossar, N., Antebi, Y., Stelzer, Y., Levanon, E.Y., and Schwartz, S. (2021). Deciphering the principles of the RNA editing code via large-scale systematic probing. Mol. Cell *81*, 2374–2387.e3.
15. Abudayyeh, O.O., Gootenberg, J.S., Franklin, B., Koob, J., Kellner, M.J., Ladha, A., Joung, J., Kirchgatterer, P., Cox, D.B.T., and Zhang, F. (2019). A cytosine deaminase for programmable single-base RNA editing. Science *365*, 382–386.
16. Montiel-Gonzalez, M.F., Vallecillo-Viejo, I.C., and Rosenthal, J.J.C. (2016). An efficient system for selectively altering genetic information within mRNAs. Nucleic Acids Res. *44*, e157.
17. Katrekar, D., Chen, G., Meluzzi, D., Ganesh, A., Worlikar, A., Shih, Y.-R., Varghese, S., and Mali, P. (2019). In vivo RNA editing of point mutations via RNA-guided adenosine deaminases. Nat. Methods *16*, 239–242.
18. Azad, M.T.A., Bhakta, S., and Tsukahara, T. (2017). Site-directed RNA editing by adenosine deaminase acting on RNA for correction of the genetic code in gene therapy. Gene Ther. *24*, 779–786.
19. Azad, M.T.A., Qulsum, U., and Tsukahara, T. (2019). Comparative Activity of Adenosine Deaminase Acting on RNA (ADARs) Isoforms for Correction of Genetic Code in Gene Therapy. Curr. Gene Ther. *19*, 31–39.

20. Cox, D.B.T., Gootenberg, J.S., Abudayyeh, O.O., Franklin, B., Kellner, M.J., Joung, J., and Zhang, F. (2017). RNA editing with CRISPR-Cas13. Science *358*, 1019–1027.

21. Marina, R.J., Brannan, K.W., Dong, K.D., Yee, B.A., and Yeo, G.W. (2020). Evaluation of Engineered CRISPR-Cas-Mediated Systems for Site-Specific RNA Editing. Cell Rep. *33*, 108350.

22. Vogel, P., Moschref, M., Li, Q., Merkle, T., Selvasaravanan, K.D., Li, J.B., and Stafforst, T. (2018). Efficient and precise editing of endogenous transcripts with SNAP-tagged ADARs. Nat. Methods *15*, 535–538.

23. Hanswillemenke, A., Kuzdere, T., Vogel, P., Jékely, G., and Stafforst, T. (2015). Site-Directed RNA Editing in Vivo Can Be Triggered by the Light-Driven Assembly of an Artificial Riboprotein. J. Am. Chem. Soc. *137*, 15875–15881.

24. Stroppel, A.S., Lappalainen, R., and Stafforst, T. (2021). Controlling Site-Directed RNA Editing by Chemically Induced Dimerization. Chem.–Eur. J. *27*, 12300–12304.

25. Stroppel, A.S., Latifi, N., Hanswillemenke, A., Tasakis, R.N., Papavasiliou, F.N., and Stafforst, T. (2021). Harnessing self-labeling enzymes for selective and concurrent A-to-I and C-to-U RNA base editing. Nucleic Acids Res. *49*, e95.

26. Stafforst, T., and Schneider, M.F. (2012). An RNA-deaminase conjugate selectively repairs point mutations. Angew. Chem. *51*, 11166–11169.

27. Matthews, M.M., Thomas, J.M., Zheng, Y., Tran, K., Phelps, K.J., Scott, A.I., Havel, J., Fisher, A.J., and Beal, P.A. (2016). Structures of human ADAR2 bound to dsRNA reveal base-flipping mechanism and basis for site selectivity. Nat. Struct. Mol. Biol. *23*, 426–433.

28. Kuttan, A., and Bass, B.L. (2012). Mechanistic insights into editing-site specificity of ADARs. Proc. Natl. Acad. Sci. USA *109*, E3295–E3304.

29. Valegard, K., Murray, J.B., Stockley, P.G., Stonehouse, N.J., and Liljas, L. (1994). Crystal structure of an RNA bacteriophage coat protein-operator complex. Nature *371*, 623–626.

30. Ferry, Q.R.V., Lyutova, R., and Fulga, T.A. (2017). Rational design of inducible CRISPR guide RNAs for de novo assembly of transcriptional programs. Nat. Commun. *8*, 14633.

31. Peng, Y., Shu, L., Deng, X., Huang, X., Mo, X., Du, F., and Tang, Z. (2023). Live-Cell Imaging of Endogenous RNA with a Genetically Encoded Fluorogenic Allosteric Aptamer. Anal. Chem. *95*, 13762–13768.

32. Li, Y., Teng, X., Zhang, K., Deng, R., and Li, J. (2019). RNA Strand Displacement Responsive CRISPR/Cas9 System for mRNA Sensing. Anal. Chem. *91*, 3989–3996.

33. Liu, Y., Zhan, Y., Chen, Z., He, A., Li, J., Wu, H., Liu, L., Zhuang, C., Lin, J., Guo, X., et al. (2016). Directing cellular information flow via CRISPR signal conductors. Nat. Methods *13*, 938–944.

34. Lin, B., An, Y., Meng, L., Zhang, H., Song, J., Zhu, Z., Liu, W., Song, Y., and Yang, C. (2019). Control of CRISPR-Cas9 with small molecule-activated allosteric aptamer regulating sgRNAs. Chem. Commun. *55*, 12223–12226.

35. Montiel-Gonzalez, M.F., Vallecillo-Viejo, I., Yudowski, G.A., and Rosenthal, J.J.C. (2013). Correction of mutations within the cystic fibrosis transmembrane conductance regulator by site-directed RNA editing. Proc. Natl. Acad. Sci. USA *110*, 18285–18290.

36. Sinnamon, J.R., Kim, S.Y., Corson, G.M., Song, Z., Nakai, H., Adelman, J.P., and Mandel, G. (2017). Site-directed RNA repair of endogenous Mecp2 RNA in neurons. Proc. Natl. Acad. Sci. USA *114*, E9395–E9402.

37. Vallecillo-Viejo, I.C., Liscovitch-Brauer, N., Montiel-Gonzalez, M.F., Eisenberg, E., and Rosenthal, J.J.C. (2018). Abundant off-target edits from site-directed RNA editing can be reduced by nuclear localization of the editing enzyme. RNA Biol. *15*, 104–114.

38. Yuan, J., Wang, X., Zhang, Y., Hu, X., Deng, X., Fei, J., and Li, N. (2006). ShRNA transcribed by RNA pol II promoter induce RNA interference in mammalian cell. Mol. Biol. Rep. *33*, 43–49.

39. Xie, K., Minkenberg, B., and Yang, Y. (2015). Boosting CRISPR/Cas9 multiplex editing capability with the endogenous tRNA-processing system. Proc. Natl. Acad. Sci. USA *112*, 3570–3575.

40. Katrekar, D., Xiang, Y., Palmer, N., Saha, A., Meluzzi, D., and Mali, P. (2022). Comprehensive interrogation of the ADAR2 deaminase domain for engineering enhanced RNA editing activity and specificity. Elife *11*, e75555.

41. Liu, Y., Mao, S., Huang, S., Li, Y., Chen, Y., Di, M., Huang, X., Lv, J., Wang, X., Ge, J., et al. (2020). REPAIRx, a specific yet highly efficient programmable A > I RNA base editor. EMBO J. *39*, e104748.

OMTN, Volume *36*

Supplemental information

Enhancing RNA editing efficiency and specificity with engineered ADAR2 guide RNAs

Xilei Ai, Sheng Ding, Shan Zhou, Feng Du, Shuai Liu, Xin Cui, Juan Dong, Xin Huang, and Zhuo Tang

Table S1: Guide sequence region of gRNAs.

Name	Sequence
1MS2-gRNA-dGFP	**cacgagggtgggcCagggcacg**ACATGAGGATCACCCATGT
1MS2-gRNA-dGFP (13nt blocker)	**GTGATCCTCATGTcacgagggtgggcCagggcacg**ACATGAGGATCACCCATGT
1MS2-gRNA-dGFP (15nt blocker)	**GGGTGATCCTCATGTcacgagggtgggcCagggcacg**ACATGAGGATCACCCATGT
1MS2-gRNA-dGFP (17nt blocker)	**ATGGGTGATCCTCATGTcacgagggtgggcCagggcacg**ACATGAGGATCACCCATGT
1MS2-gRNA-dGFP (19nt blocker)	**ACATGGGTGATCCTCATGTcacgagggtgggcCagggcacg**ACATGAGGATCACCCATGT
1MS2-gRNA-dGFP (21nt blocker)	**ACATGGGTGATCCTCATGTCGcacgagggtgggcCagggcacg**ACATGAGGATCACCCATGT
1MS2-gRNA-dGFP (23nt blocker)	**ACATGGGTGATCCTCATGTCGTGcacgagggtgggcCagggcacg**ACATGAGGATCACCCATGT
1MS2-gRNA-dGFP (25nt blocker)	**ACATGGGTGATCCTCATGTCGTGCCcacgagggtgggcCagggcacg**ACATGAGGATCACCCATGT
BSM-gRNA （15nt）-dGFP	**ACATGGGTGATCCTCATGTggtgggcCagggcac**ACATGAGGATCACCCATGT
BSM-gRNA （21nt）-dGFP	**ACATGGGTGATCCTCATGTgagggtgggcCagggcacggg**ACATGAGGATCACCCATGT
BSM-gRNA （25nt）-dGFP	**ACATGGGTGATCCTCATGTacgagggtgggcCagggcacgggca**ACATGAGGATCACCCATGT
BSM-gRNA （31nt）-dGFP	**ACATGGGTGATCCTCATGTgtcacgagggtgggcCagggcacgggcagct**ACATGAGGATCACCCATGT
1BoxB-gRNA-dGFP	**cacgagggtgggcCagggcacgGGCCCTGAAAAAGGGCC**
BSB-gRNA-dGFP (19nt blocker)	**ACATGGGTGATCCTCATGTcacgagggtgggcCagggcacgGGCCCTGAAAAAGGGCC**

1BoxB-gRNA- dGFP (2 mismatches)	**acgagggtaggcCagggtacgggcaGGCCCTGAAAAAGGGCC**
1BoxB-gRNA- dGFP (4 mismatches)	**acgagcgtaggcCagggtaccggcaGGCCCTGAAAAAGGGCC**
BSB-gRNA-dGFP (2 mismatches)	**ACATGGGTGATCCTCATGTacgagggtaggcCagggtacgggcaGGCCCTGAAAAAGGGCC**
BSB-gRNA-dGFP (4mismatches)	**ACATGGGTGATCCTCATGTacgagcgtaggcCagggtaccggcaGGCCCTGAAAAAGGGCC**
1BoxB-gRNA-ACTIN	**CctctcttgctcCgggcctcgtcgcGGCCCTGAAAAAGGGCC**
BSB-gRNA-ACTIN	**GGCCCTTTTTCAGGGCCGC**cctctcttgctcCgggcctcgtcgc**GGCCCTGAAAAAGGGCC**
1BoxB-gRNA-ACTIN (TCG)	**CcagttggtgacTatgccgtgctcgGGCCCTGAAAAAGGGCC**
BSB-gRNA-ACTIN (TCG)	**GGCCCTTTTTCAGGGCCCGccagttggtgacTatgccgtgctcgGGCCCTGAAAAAGGGCC**
1BoxB-gRNA-TYMS (TCG)	**TttcagtggctcTatgtgattcaggGGCCCTGAAAAAGGGCC**
BSB-gRNA-TYMS (TCG)	**GGCCCTTTTTCAGGGCCCCTttcagtggctcTatgtgattcaggGGCCCTGAAAAAGGGCC**

Table S2: Sequences of Constructs Used in this Study.

Name	Sequence
dGFP	ATGGTGAGCAAGGGCGAGGAGCTGTTCACCGGGGTGGTGCCCATCCTGGTCGAGCTGGACGGCGACGTAAACGGCCACAAGTTCAGCGTGTCCGGCGAGGGCGAGGGCGATGCCACCTACGGCAAGCTGACCCTGAAGTTCATCTGCACCACCGGCAAGCTGCCCGTGCCC**TAG**CCCACCCTCGTGACCACCCTGACCTACGGCGTGCAGTGCTTCAGCCGCTACCCCGACCACATGAAGCAGCACGACTTCTTCAAGTCCGCCATGCCCGAAGGCTACGTCCAGGAGCGCACCATCTTCTTCAAGGACGACGGCAACTACAAGACCCGCGCCGAGGTGAAGTTCGAGGGCGACACCCTGGTGAACCGCATCGAGCTGAAGGGCATCGACTTCAAGGAGGACGGCAACATCCTGGGGCACAAGCTGGAGTACAACTACAACAGCCACAACGTCTATATCATGGCCGACAAGCAGAAGAACGGCATCAAGGTGAACTTCAAGATCCGCCACAACATCGAGGACGGCAGCGTGCAGCTCGCCGACCACTACCAGCAGAACACCCCCATCGGCGACGGCCCCGTGCTGCT

	GCCCGACAACCACTACCTGAGCACCCAGTCCGCCCTGAG<br>CAAAGACCCCAACGAGAAGCGCGATCACATGGTCCTGCT<br>GGAGTTCGTGACCGCCGCCGGGATCACTCTCGGCATGGA<br>CGAACTGTACAAGTAA
MCP-ADAR-NES	ATGGCTTCTAACTTTACGCAGTTTGTGCTCGTCGATAATG<br>GGGGAACAGGGGACGTGACAGTTGCCCCCTCAAACTTTG<br>CCAACGGCGTCGCTGAGTGGATTTCATCTAATTCCCGAAG<br>TCAGGCCTACAAGGTCACCTGCTCTGTTAGGCAAAGTTCA<br>GCCCAGAACCGCAAGTACACCATAAAGGTTGAAGTGCCC<br>AAGGTCGCGACCCAGACAGTCGGCGGAGTCGAACTGCCT<br>GTGGCTGCTTGGCGCTCATATCTCAATATGGAACTTACAA<br>TCCCGATCTTTGCAACTAATTCCGATTGTGAATTGATAGT<br>TAAGGCAATGCAGGGCTTGCTGAAGGATGGAAATCCGAT<br>CCCTTCAGCAATTGCCGCCAATAGCGGGATCTACGGATCC<br>GGAGGAGGTGGAAGCcagctgcatttaccgcaggttttagctgacgctgtctcac<br>gcctggtcctgggtaagtttggtgacctgaccgacaacttctcctcccctcacgctcgcagaaaa<br>gtgctggctggagtcgtcatgacaacaggcacagatgttaaagatgccaaggtgataagtgttt<br>ctacaggaacaaaatgtattaatggtgaatacatgagtgatcgtggccttgcattaaatgactgcc<br>atgcagaaataatatctcggagatccttgctcagatttctttatacacaacttgagctttacttaaata<br>acaaagatgatcaaaaaagatccatctttcagaaatcagagcgaggggggtttaggctgaagg<br>agaatgtccagtttcatctgtacatcagcacctctccctgtggagatgccagaatcttctcaccac<br>atgagccaatcctggaagaaccagcagatagacacccaaatcgtaaagcaagaggacagcta<br>cggaccaaaatagagtctggtCaggggacgattccagtgcgctccaatgcgagcatccaaac<br>gtgggacggggtgctgcaaggggagcggctgctcaccatgtcctgcagtgacaagattgcac<br>gctggaacgtggtgggcatccagggatcActgctcagcattttcgtggagcccatttacttctcg<br>agcatcatcctgggcagcctttaccacggggaccacctttccagggccatgtaccagcggatct<br>ccaacatagaggacctgccacctctctacaccctcaacaagcctttgctcagtggcatcagcaat<br>gcagaagcacggcagccagggaaggcccccaacttcagtgtcaactggacggtaggcgact<br>ccgctattgaggtcatcaacgccacgactgggaaggatgagctgggccgcgcgtcccgcctg<br>tgtaagcacgcgttgtactgtcgctggatgcgtgtgcacggcaaggttccctcccacttactacg<br>ctccaagattaccaagcccaacgtgtaccatgagtccaagctggcggcaaaggagtaccagg<br>ccgccaaggcgcgtctgttcacagccttcatcaaggcggggctgggggcctgggtggagaag<br>cccaccgagcaggaccagttctcactcacgGGGAGCGGATCCGGCTCTCT<br>GCCACCATTGGAAAGGCTTACGCTGTAA
4λN-ADAR-NES[1]	ATGGCGAACGCCAGAACCCGACGCAGAGAGAGGAGGGC<br>AGAGAAACAGGCCCAGTGGAAAGCCGCTAACGGAGGAG<br>GGGGATCCGGTGGAGGCGGCAGTGGAGGCGGGGGATCTA<br>ATGCGCGGACCCGCCGACGTGAACGGCGAGCAGAAAAAC<br>AGGCGCAATGGAAAGCAGCCAACGGCGGCGGTGGAAGT<br>GGAGGTGGTGGAAGCGGAGGCGGTGGTAGTAATGCCCGT<br>ACGCGCCGGCGCGAACGGCGAGCAGAAAAGCAGGCACA<br>GTGGAAGGCAGCTAATGGCGGAGGGGGAAGCGGGGGCG

	GTGGAAGTGGGGGTGGAGGCAGCAACGCACGAACACGA<br>CGACGTGAGCGTCGCGCTGAGAAACAAGCTCAATGGAAA<br>GCTGCAAACGGATCCGGAGGAGGTGGAAGCcagctgcatttaccg<br>caggttttagctgacgctgtctcacgcctggtcctgggtaagtttggtgacctgaccgacaacttc<br>tcctcccctcacgctcgcagaaaagtgctggctggagtcgtcatgacaacaggcacagatgtta<br>aagatgccaaggtgataagtgtttctacaggaacaaaatgtattaatggtgaatacatgagtgatc<br>gtggccttgcattaaatgactgccatgcagaaataatatctcggagatccttgctcagatttctttat<br>acacaacttgagctttacttaaataacaaagatgatcaaaaaagatccatctttcagaaatcagag<br>cgaggggggtttaggctgaaggagaatgtccagtttcatctgtacatcagcacctctccctgtgg<br>agatgccagaatcttctcaccacatgagccaatcctggaagaaccagcagatagacacccaaa<br>tcgtaaagcaagaggacagctacggaccaaaatagagtctggtCaggggacgattccagtgc<br>gctccaatgcgagcatccaaacgtgggacggggtgctgcaaggggagcggctgctcaccat<br>gtcctgcagtgacaagattgcacgctggaacgtggtgggcatccagggatcActgctcagcat<br>tttcgtggagcccatttacttctcgagcatcatcctgggcagcctttaccacggggaccacctttc<br>cagggccatgtaccagcggatctccaacatagaggacctgccacctctctacaccctcaacaa<br>gcctttgctcagtggcatcagcaatgcagaagcacggcagccagggaaggcccccaacttca<br>gtgtcaactggacggtaggcgactccgctattgaggtcatcaacgccacgactgggaaggatg<br>agctgggccgcgcgtcccgcctgtgtaagcacgcgttgtactgtcgctggatgcgtgtgcacg<br>gcaaggttccctcccacttactacgctccaagattaccaagcccaacgtgtaccatgagtccaa<br>gctggcggcaaaggagtaccaggccgccaaggcgcgtctgttcacagccttcatcaaggcgg<br>ggctgggggcctgggtggagaagcccaccgagcaggaccagttctcactcacgGGGAG<br>CGGATCCGGCTCTCTGCCACCATTGGAAAGGCTTACGCTG
4λN-ADAR$_{\text{RECUSE}}$-NES^{2} ADAR$_{\text{RESCUE}}$ contains mutations of ADAR2(E488Q/V351G/S486A/T375S/S370C/P462A/N597I/L332I/I398V/K350I/M383L/D619G/S582T/ V440I/ S495N/ K418E	ATGGCGAACGCCAGAACCCGACGCAGAGAGAGGAGGGC<br>AGAGAAACAGGCCCAGTGGAAAGCCGCTAACGGAGGAG<br>GGGGATCCGGTGGAGGCGGCAGTGGAGGCGGGGGATCTA<br>ATGCGCGGACCCGCCGACGTGAACGGCGAGCAGAAAAAC<br>AGGCGCAATGGAAAGCAGCCAACGGCGGCGGTGGAAGT<br>GGAGGTGGTGGAAGCGGAGGCGGTGGTAGTAATGCCCGT<br>ACGCGCCGGCGCGAACGGCGAGCAGAAAAGCAGGCACA<br>GTGGAAGGCAGCTAATGGCGGAGGGGGAAGCGGGGGCG<br>GTGGAAGTGGGGGTGGAGGCAGCAACGCACGAACACGA<br>CGACGTGAGCGTCGCGCTGAGAAACAAGCTCAATGGAAA<br>GCTGCAAACGGATCCGGAGGAGGTGGAAGCcagctgcatttaccg<br>caggttttagctgacgctgtctcacgcCTGgtcATAggtaagtttggtgacctgaccgaca<br>acttctcctcccctcacgctcgcagaaTaGGTctggctggagtcgtcatgacaacaggcaca<br>gatgttaaagatgccAAGgtgataTGTgtttctacaggaTCTaaatgtattaatggtgaat<br>acCTAagtgatcgtggccttgcattaaatgactgccatgcagaaataGtatctcggagatcctt<br>gctcagatttctttatacacaacttgagctttacttaaataacGAGgatgatcaaaaaagatccat<br>ctttcagaaatcagagcgaggggggtttaggctgaaggagaatATAcagtttcatctgtacat<br>cagcacctctccctgtggagatgccagaatcttctcaccacatgagGCAatcctggaagaac<br>cagcagatagacacccaaatcgtaaagcaagaggacagctacggaccaaaatagagGCTg<br>gtCaggggacgattccaGTGcgcAACaatgcgagcatccaaacgtgggacggggtgct<br>gcaaggggagcggCTGctcaccatgtcctgcagtgacaagattgcacgctggaacgtggtg

ggcatccagggatcActgctcagcattttcgtggagcccatttacttctcgagcatcatcctggg
cagcctttaccacggggaccaccttTCCagggccatgtacCAGcggatctccaacataga
ggacctgccacctctctacaccctcaacaagcctttgctcACAggcatcagcaatgcagaag
cacggcagccagggaaggcccccATAttcagtgtcaactggacggtaggcgactccgcta
ttgaggtcatcaacgccacgactgggaagGGAGAGctgggccgcgcgtcccgcctgtgt
aagcacgcgttgtactgtcgctggatgcgtgtgcacggcaaggttccctcccacttaCTAcgc
tccaagattaccaagcccaacgtgtaccatgagACAaagctggcggcaaaggagtaccag
gccgccaaggcgcgtctgttcacagccttcatcaaggcggggctgggggcctgggtggagaa
gcccaccgagcaggaccagttctcactcacgGGGAGCGGATCCGGCTCTCT
GCCACCATTGGAAAGGCTTACGCTGTAA

Table S3: PCR, and Sequencing primers.

Name	Sequence
GFP-PCR-F	GGGCGAGGAGCTGTTCACC
GFP-PCR-R	GCTGCCGTCCTCGATGTTG
GAPDH-PCR-F	GAAATCCCATCACCATCTTCC
GAPDH-PCR-R	GGTCTCTCTCTTCCTCTTGT
Actin-PCR-F	ATGGATGATGATATCGCCGC
Actin-PCR-R	GAGGCGTACAGGGATAGCACA
TYMS-PCR-F	CAGGACAGGGAGTTGACCAA
TYMS-PCR-R	GTCTTTAGGGGTTGGGCTGG

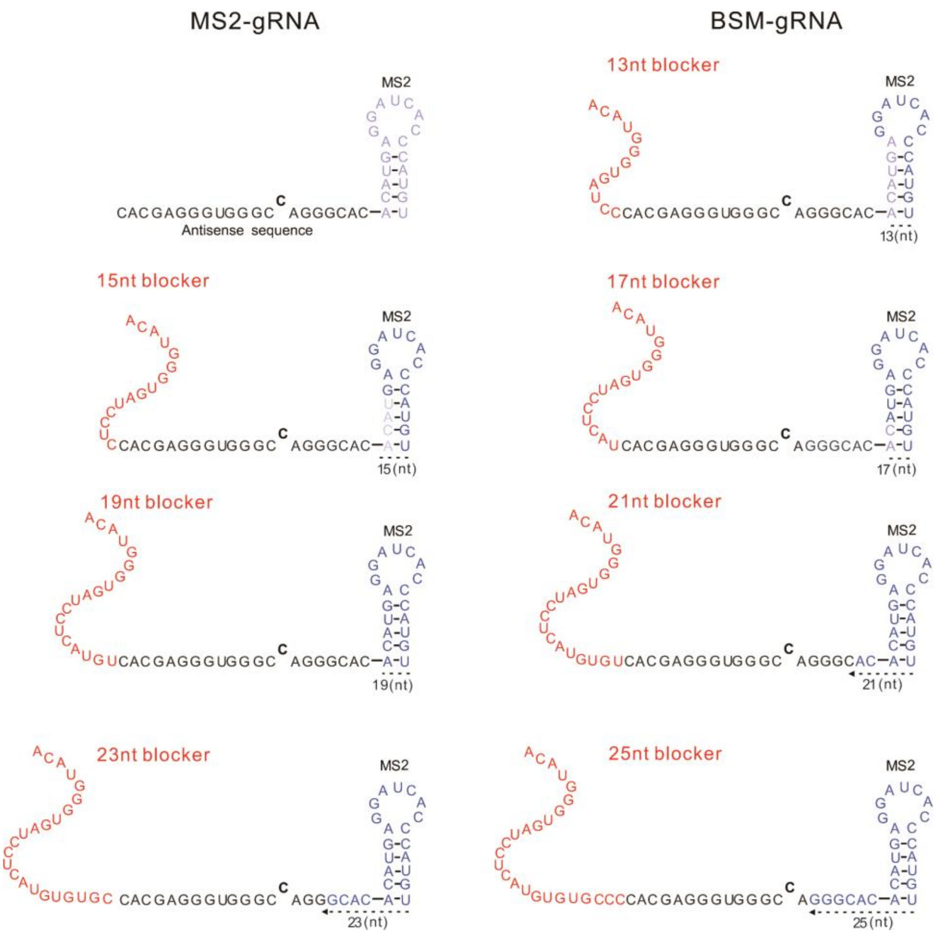

Figure S1. The schematic diagram of MS2-gRNA and BSM-gRNA with different blocking sequence lengths. As shown in the figure below, all blocking sequences (red) and their complementary sequences (blue) used in the BSM-gRNA design are displayed. When the blocking sequence exceeded the length of the MS2 hairpin (19 nt), extending the blocking sequence by targeting regions near the MS2 hairpin, gradually elongating it from the 3' to the 5' end. This design was also applied to the BoxB system.

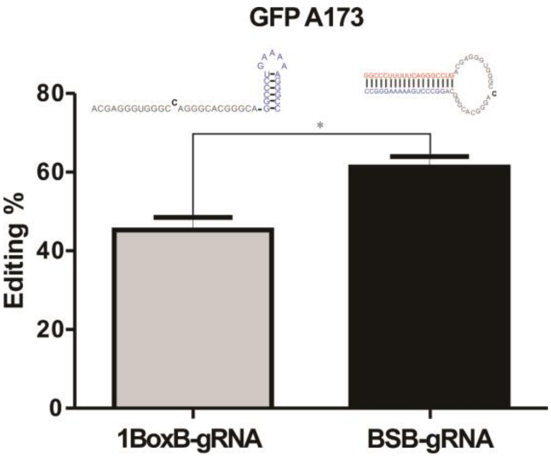

Figure S2. 1BoxB-gRNA and BSB-gRNA mediated editing of dGFP A173. ADAR-mediated editing efficiencies with the addition of plasmids expressing 1BoxB-gRNA, BSB-gRNA that target the same site (dead-GFP). HEK293 cells were cultured in 24-well plates. 500ng of pcDNA3.1-1BoxB-λN-ADAR (or pcDNA3.1-BSB-λN-ADAR) and 500 ng pcDNA3.1-dead GFP report plasmids were transfected when cells grow to approximately 70–80% confluence. All transfections were conducted using Hieff Tran^{TM} Liposomal Transfection Reagent (Yeasen) according to the manufacturer's recommendations. The transformed media was changed at 24 hours and the cells were incubated for additional 48 hours before analysis. Finally, the targeted RNA editing was confirmed by RT-PCR/Sanger sequencing. The blocking sequence containing BoxB-gRNA was paired with the target RNA of dead-GFP, and the results showed a 1.4-fold increase in BSB-gRNA over BoxB-gRNA to 61%. All values are mean ± SEM with n = 3 biological replicates. Student t-test: **P* <0.05, ***P* <0.01, ****P* <0.001.

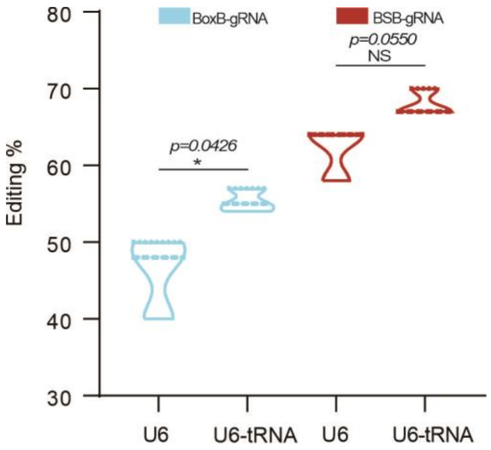

Figure S3. Effect of U6 and U6-tRNA promoters on RNA editing efficiency in the SPRING system using BoxB-gRNA and BSB-gRNA. The figure shows the RNA editing efficiencies of BoxB-gRNA and BSB-gRNA driven by U6 and U6-tRNA promoters in the SPRING system. The U6-tRNA promoter significantly improved editing efficiency for BoxB-gRNA, increasing from 62% (U6) to 68% (P = 0.0426, * indicates statistical significance). However, for BSB-gRNA, the increase was not statistically significant (P = 0.0550, NS indicates not significant).

Note: Solid lines represent the median, and dashed lines denote the first and third quartiles. Statistical analysis was performed using a two-tailed Student's t-test..

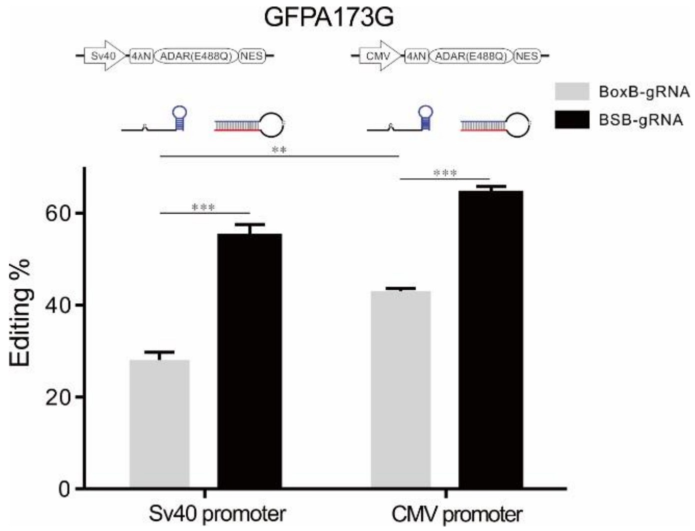

Figure S4. **The SV40 promoter and the CMV promoter respectively mediate RNA editing.** To further validate this mechanism, we transcribed the effector 4λN-ADAR(E488Q) using two different promoters (SV40 and CMV). ADAR-mediated editing efficiencies with the addition of plasmids expressing 1BoxB-gRNA, BSB-gRNA that target the same site (dead-GFP). HEK293 cells were cultured in 24-well plates. 500ng of pcDNA3.1-1BoxB-λN-ADAR (or pcDNA3.1-BSB-λN-ADAR) and 500 ng pcDNA3.1-dead GFP report plasmids were transfected when cells grow to approximately 70–80% confluence. All transfections were conducted using Hieff Tran^{TM} Liposomal Transfection Reagent (Yeasen) according to the manufacturer's recommendations. The transformed media was changed at 24 hours and the cells were incubated for additional 48 hours before analysis. Finally, the targeted RNA editing was confirmed by RT-PCR/Sanger sequencing. The editing efficiency of dGFP 173A site BoxB-gRNA transcribed by the SV40 promoter was 28%, compared to 55% for 1BoxB-gRNA, representing a 49% increase. For the CMV promoter-transcribed effector, BoxB-gRNA editing efficiency was 64%, compared to 43% for 1BoxB-gRNA, resulting in a 33% improvement. All values are mean ± SEM with n = 3 biological replicates. Student t-test: **P* <0.05, ***P* <0.01, ****P* <0.001.

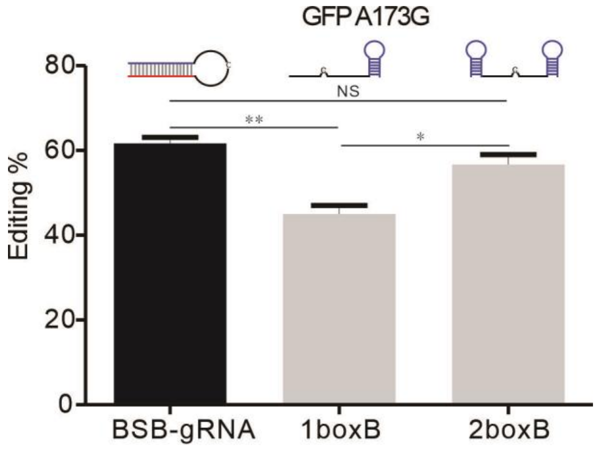

Figure S5. **Comparison of SPRING system with different numbers of BoxB guides.** Furthermore, we conducted a comparative analysis of RNA editing utilizing our system alongside the guide RNA containing two BoxB aptamers positioned towards both termini of the antisense part. HEK293 cells were cultured in 24-well plates. 500ng of pcDNA3.1-1BoxB-λN-ADAR (or pcDNA3.1-BSB-λN-ADAR) and 500 ng pcDNA3.1-dead GFP report plasmids were transfected when cells grow to approximately 70–80% confluence. All transfections were conducted using Hieff Tran^{TM} Liposomal Transfection Reagent (Yeasen) according to the manufacturer's recommendations. The transformed media was changed at 24 hours and the cells were incubated for additional 48 hours before analysis. Finally, the targeted RNA editing was confirmed by RT-PCR/Sanger sequencing.
At the dGFP 173A site, the editing efficiency of 2BoxB-gRNA was 57%, higher than the 45% efficiency of 1BoxB-gRNA, while the editing efficiency of BSB-gRNA at this site was 62%, surpassing the editing efficiencies of both at this location. All values are mean ± SEM with n = 3 biological replicates. Using one-way ANOVA revealed significant differences among the three groups (F=18.99,P=0.0025,R^2=0.8636). Tukey's post hoc test was performed to identify pairwise differences: BSB-gRNA *vs.* 1BoxB-gRNA (*P*=0.0023, ＊＊), BSB-gRNA *vs.* 2BoxB-gRNA (*P*=0.2473,ns), 1BoxB-gRNA *vs.* 2BoxB-gRNA (*P*=0.0134,*). Significance thresholds: **P*<0.05,** *P*<0.01,****P*<0.001.

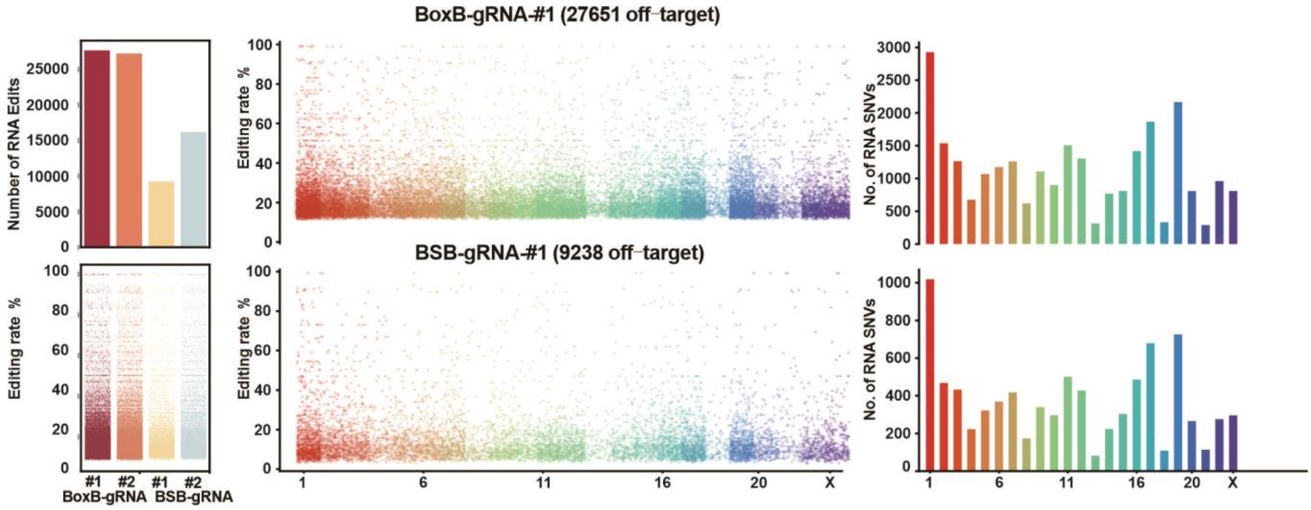

Figure S6. **Analysis of transcriptome-wide off-target editing in the BoxB-gRNA group and BSB-gRNA group.** Furthermore, we sought to evaluate the specificity of BSB-gRNAs both at the transcriptome-wide level and for target transcripts. HEK293T cells were cultured in 6-well plates, and 2500 ng of pcDNA3.1-4λN-DD-1BoxB-gRNA (or pcDNA3.1-4λN-DD-BSB-gRNA) plasmids were transfected when cells reached approximately 70–80% confluence. All transfections were performed using Hieff-Tran™ Liposomal Transfection Reagent (Yeasen), following the manufacturer's instructions. The media were replaced after 24 hours, and the cells were incubated for an additional 48 hours prior to analysis. To assess transcriptome-wide specificity, deep RNA-seq analysis was conducted on samples transfected with BoxB-gRNA or BSB-gRNA, as well as an untransfected HEK293T control. The results revealed that compared to BoxB-gRNA, which exhibited nearly 30,000 off-target sites, BSB-gRNA demonstrated a substantial reduction, with approximately 12,000 off-target sites. These findings highlight the enhanced efficiency and specificity of the SPRING system, surpassing the performance of the original BoxB-λN-ADAR system ($n=2$; $p=0.0259$; unpaired t-test, one-tailed).

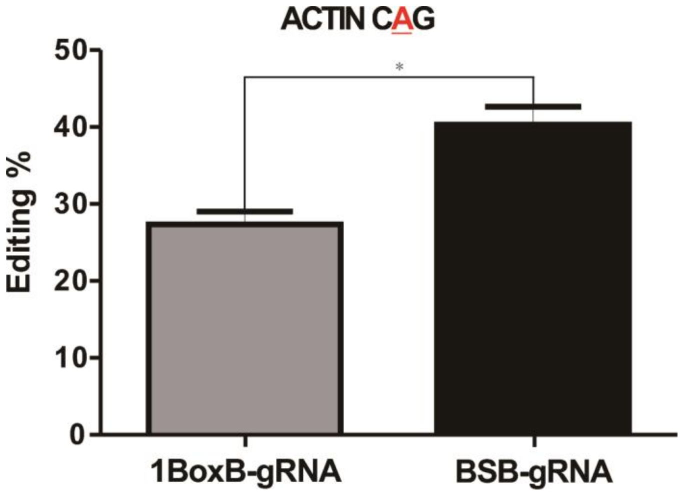

Figure S7. **1BoxB-gRNA and BSB-gRNA mediated editing of CAG site in the ORF of ACTIN.** We next sought to assess their editing efficiency in endogenous transcript editing. We selected a UAG editing site within the open reading frame (ORF) of ACTIN, which is usually the target of other RNA base editing systems. We introduced 4λNs-DD and BSB-gRNA into the cells and aligned the gRNAs along the endogenous transcripts. Analysing the editing of the endogenous RNAs by RT-PCR/Sanger sequencing, we found that the peak of editing appeared 48 hours after transfection. The level of A-I RNA editing increased from 27% to 40% at specific sites of the ACTIN gene. In conclusion, these results confirm that BSB-gRNA can effectively improve the efficiency of ADAR-mediated RNA editing. All values are mean ± SEM with n = 3 biological replicates. Student t-test: *P <0.05, **P <0.01, ***P <0.001.

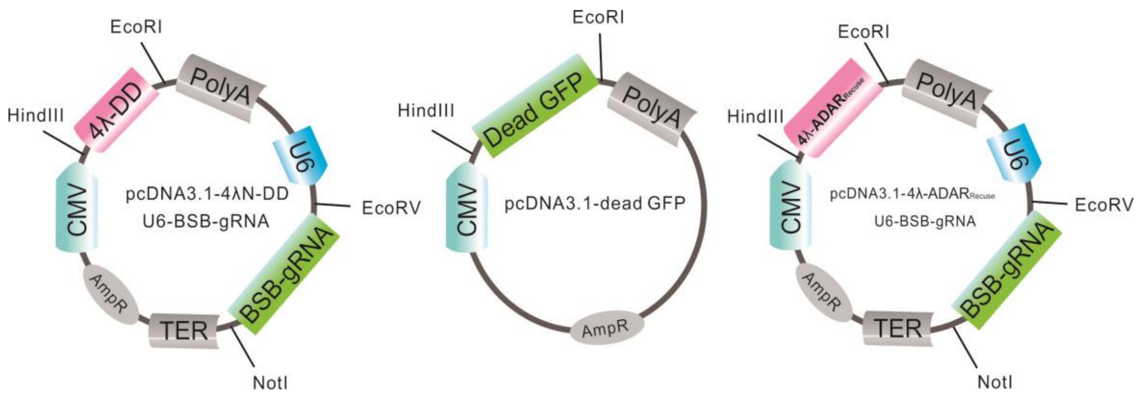

Figure S8. **The Plasmid maps used in the work.** The E. coli strains used in this study were Trelief™ 5α Chemically Competent Cells, purchased from Tsingke Biotechnology (Beijing, China). The 4λN-DD (E488Q), 4λN-ADAR$_{RESCUE}$, and Dead GFP reporter genes were cloned into a modified pcDNA3.1 zeo (+)-U6 vector at the Hind-III and Eco-RI restriction endonuclease sites. For the investigated guide RNA, it was cloned into a modified pcDNA3.1 zeo (+) vector at the Eco-RV and Not-I restriction endonuclease sites.

References

1. Montiel-Gonzalez, MF, Vallecillo-Viejo, IC, and Rosenthal, JJC (2016). An efficient system for selectively altering genetic information within mRNAs. *Nucleic Acids Research* **44**.
2. Abudayyeh, OO, Gootenberg, JS, Franklin, B, Koob, J, Kellner, MJ, Ladha, A, *et al.* (2019). A cytosine deaminase for programmable single-base RNA editing. *Science* **365**: 382-+.
